# Supplementary material for: Identification of Freezing-Responsive microRNAs and Their Targets in Chinese Jujube by Small RNA and Degradome Sequencing
Source: Int J Mol Sci. 2026 Sep 4;27(17):7912. doi: 10.3390/ijms27177912 (PMC13566890; doi:10.3390/ijms27177912)
Supplement: Supplementary file 1 [file ijms-27-07912-s001.zip › Supplementary Material S2/novel_miR_92.pdf]

Provisional ID : novel_miR_92
Score total : 229577.7
Score for star read(s) : 3.9
Score for read counts : 229571.8
Score for mfe : 0.4
Score for randfold : 1.6
Score for cons. seed :
Total read count : 450307
Mature read count : 395695
Loop read count : 20
Star read count : 54592

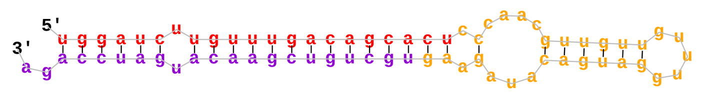

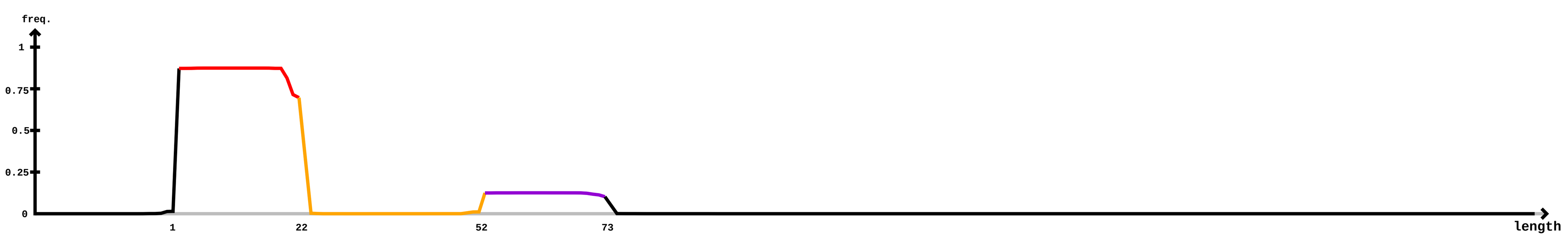

Mature Star

```
5'- cccaccucucuuguuuaccuucuggaucuuguuugacagcacucccaacguuguuguuuggaugacauagaagugcugucgaacaugauccagaaggugcugucgaacaacauacaaaaagggguuuacuuuacguaccgcccaucacgaaaaaaaaaaaaaaauuguuuaauauuauuucaagauuuauuugaaugaaaauagaacagcuauuccuugguuucuuuaauuacuaaaacccuaugaucuua -3' obs
    cccaccucucuuguuuaccuucuggaucuuguuugacagcacucccaacguuguuguuuggaugacauagaagugcugucgaacaugauccagaaggugcugucgaacaacauacaaaaagggguuuacuuuacguaccgcccaucacgaaaaaaaaaaaaaaauuguuuaauauuauuucaagauuuauuugaaugaaaauagaacagcuauuccuugguuucuuuaauuacuaaaacccuaugaucuua     exp
    ..........(((((((((((((((((.((((((((((((((.(....((((((.....))))))...).)))))))))))))).))))))))))))......))))))((((.......((((.(((.....)))..))))..................((((((.((.((..((((((.....))))))..)).)).))))))........(((((...........))))).)))).....  reads  mm  sample
    ..............uuaccuucuggaucuuguuug................................................................................................................................................................................................................  2  0  JCK-2
    ................accuucuggaucuuguuug................................................................................................................................................................................................................  2  0  LCK-3
    ................accuucuggaucuuguuuga...............................................................................................................................................................................................................  4  0  LT-2
    ................accuucuggaucuuguuuga...............................................................................................................................................................................................................  2  0  JCK-1
    ................accuucuggaucuuguuuga...............................................................................................................................................................................................................  1  0  JCK-2
    ................accuucuggaucuuguuuga...............................................................................................................................................................................................................  3  0  LT-1
    ................accuucuggaucuuguuuga...............................................................................................................................................................................................................  2  0  LT-3
    ................acNuucuggaucuuguuuga...............................................................................................................................................................................................................  1  1  LT-2
    ................accuucuggaucuuguuugaA..............................................................................................................................................................................................................  1  1  JCK-1
    ................accuucuggaucuuguuugac..............................................................................................................................................................................................................  4  0  LT-1
    ................accuucuggaucuuguuugac..............................................................................................................................................................................................................  3  0  JCK-3
    ................accuucuggaucuuguuugac..............................................................................................................................................................................................................  1  0  JCK-2
    ................accuucuggaucuuguuugac..............................................................................................................................................................................................................  2  0  LT-3
    ................accuucuggaucuuguuugaca.............................................................................................................................................................................................................  2  0  JCK-1
    ................accuucuggaucuuguuugacagc...........................................................................................................................................................................................................  1  0  JCK-3
    ................accuucuggaucuuguuugacagc...........................................................................................................................................................................................................  1  0  JCK-1
    ................accuucuggaucuuguuugacagc...........................................................................................................................................................................................................  2  0  JCK-2
    .................ccuucuggaucuuguuuga...............................................................................................................................................................................................................  6  0  LCK-3
    .................ccuucuggaucuuguuuga...............................................................................................................................................................................................................  4  0  LT-2
    .................ccuucuggaucuuguuugac..............................................................................................................................................................................................................  7  0  LT-2
    .................ccuucuggaucuuguuugac..............................................................................................................................................................................................................  1  0  LT-1
    .................ccuucuggaucuuguuugaca.............................................................................................................................................................................................................  33  0  JCK-3
    .................ccuucuggaucuuguuugCca.............................................................................................................................................................................................................  1  1  LCK-2
    .................ccuucuggaucuuguuugaca.............................................................................................................................................................................................................  37  0  LCK-3
    .................ccuucuggaucuuguuugaca.............................................................................................................................................................................................................  18  0  LCK-2
    .................ccuucuggaucuuguuugaca.............................................................................................................................................................................................................  46  0  JCK-2
    .................cNuucuggaucuuguuugaca.............................................................................................................................................................................................................  1  1  JCK-1
    .................ccuucuggaucuuguuugaca.............................................................................................................................................................................................................  37  0  JT-1
    .................ccuucuggaucuuguuugaca.............................................................................................................................................................................................................  15  0  LT-1
    .................ccuucuggaucuuguuugaca.............................................................................................................................................................................................................  25  0  LT-2
    .................ccuucuggaucuuguuugaca.............................................................................................................................................................................................................  31  0  JCK-1
    .................ccuucuggaucuuguuugaca.............................................................................................................................................................................................................  27  0  LT-3
    .................ccuucuggaucuuguuugaca.............................................................................................................................................................................................................  34  0  LCK-1
```

Mature / **Star**

```
cccaccucucuuguuuaccuucuggaucuuguuugacagcacucccaacguuguuguuuggaugacauagaagugcugucgaacaugauccagaaggugcugucgaacaacauacaaaaagggguuuacuuuacguaccgcccaucacgaaaaaaaaaaaaaauuguuuaauauuauuucaagauuuauuugaaugaaaauagaacagcuauuccuugguuucuuuaauuacuaaaacccuaugaucuua
.................ccuucuggaucuuguuugacagc        3    0   JCK-3
.................ccuucuggaucuuguuugacagc        2    0   LT-1
.................ccuucuggaucuuguuugacagcac      1    0   LT-1
.................ccuucuggaucuuguuugacagcacuccc  3    0   LCK-3
..................cuucuggaucuuguuugaAa          1    1   JCK-2
..................cuucuggaucuuguuugaca          2    0   JCK-3
..................cuucuggaucuuguuugaca          2    0   LT-3
..................cuucuggaucuuguuugaca          4    0   JCK-2
..................cuucuggaucuuguuugacU          1    1   LT-3
..................cuucuggaucuuguuugaca          1    0   JCK-1
..................cuucuggaucuuguuugaca          8    0   LCK-3
..................cuucuggaucuuguuugaca          5    0   LT-2
..................cuucuggaucuuguuugacagc        1    0   JCK-3
..................cuucuggaucuuguuugacagc        2    0   JCK-1
..................cuucuggaucuuguuugacagc        2    0   LT-3
..................cuucuggaucuuguuugacagc        4    0   JCK-2
..................cuucuggaucuuguuugacagca       1    0   JCK-3
..................cuucuggaucuuguuugacagca       1    0   JCK-2
..................cuucuggaucuuguuugacagca       2    0   JCK-1
...................uucuggaucuuguuugac           6    0   LT-2
...................uucuggaucuuguuugaca          3    0   JCK-3
...................uucuggaucuuguuugaca          1    0   LCK-3
...................uucuggaucuuguuugaca          1    0   LT-2
...................uucuggaucuuguuugacU          9    1   JT-1
...................uucuggaucuuguuugaca          11   0   JT-1
...................uucuggaucuuguuugaca          1    0   JCK-1
...................uucuggaucuuguuugaca          5    0   LT-3
...................uucuggaucuuguuugacaU         1    1   LT-3
...................uucuggaucuuguuugacag         2    0   LT-2
...................uucuggaucuuguuugacagc        39   0   JCK-3
...................uucuggaucuuguuugacagc        43   0   LCK-1
...................uucuggaucuuguuugacagc        129  0   LT-1
...................uucuggaucuuguuugacagc        139  0   LT-3
...................uucuggCucuuguuugacagc        1    1   LT-1
...................uucuggCucuuguuugacagc        1    1   LT-2
...................uucuggaucuuguuugacCgc        1    1   LT-1
...................uucuggaucuuguuugacagc        131  0   LCK-3
...................uucuggaucuugCuugacagc        1    1   LCK-3
...................uucuggaCcuuguuugacagc        1    1   LT-1
...................uucugAaucuuguuugacagc        1    1   LT-1
...................uucuggaucuuguuugacagc        43   0   JCK-2
...................uucuggaucuuguuugacagc        38   0   LCK-2
...................uucuggaucuuguuugacagN        1    1   JCK-1
...................uucuggaucuuguuugacagc        29   0   JCK-1
...................uucuggaucuuguuugacagc        105  0   LT-2
...................uucuggaucuuguuugacagcU       3    1   LT-3
...................uucuggaucuuguuugacagcU       4    1   LCK-3
...................uucuggaucuuguuugacagca       1    0   JCK-3
...................uucuggaucuuguuugacagcU       5    1   LT-1
...................uucuggaucuuguuugacagcG       1    1   LT-3
...................uucuggaucuuguuugacagcU       7    1   LT-2
...................uucuggaucuuguuugacagcacu     3    0   LCK-3
....................ucuggaucuuguuugaca          9    0   JT-1
....................ucuggaucuuguuugaca          41   0   LT-2
....................ucuggaucuuguuugaca          35   0   JT-3
....................ucuggaucuuguuugaca          15   0   JCK-3
....................ucuggaucuuguuugacU          9    1   LCK-3
....................ucuggaucuuguuugaca          25   0   JCK-1
....................ucuggaucuuguuugaca          3    0   LCK-2
....................ucuggaucuuguuugaca          79   0   LCK-3
....................ucuggaucuuguuugaca          28   0   LT-1
....................ucuggaucuuguuugacU          2    1   LT-3
....................ucuggaucuuguuugacU          1    1   JCK-2
....................ucuggaucuuguuugaca          30   0   JT-2
....................ucuggaucuuguuugacU          6    1   LT-1
....................ucuggaucuuguuugaca          26   0   LT-3
....................ucuggaucuuguuugaca          3    0   LCK-1
....................ucuggaucuuguuugacU          1    1   JCK-1
....................ucuggaucuuguuugaca          33   0   JCK-2
....................ucuggaucuuguuugacag         2    0   LT-1
```

```
                    Mature                                               Star
cccaccucucuuguuuaccuucuggaucuuguuugacagcacucccaacguuguuguuuggaugacauagaagugcugucgaacaugauccagaaggugcugucgaacaacauacaaaaagggguuuacuuuacguaccgcccaucacgaaaaaaaaaaaaaaauuguuuaauauuauuucaagauuuauuugaaugaaaauagaacagcuauuccuugguuucuuuaauuacuaaaacccuaugaucuua
....................ucuggaucuuguuugacagc....................................................................................................................................................................................................   63     0    LCK-1
....................ucuggaucuuguuugacagc....................................................................................................................................................................................................   107    0    LT-1
....................ucuggaucuuguuugacagc....................................................................................................................................................................................................   29     0    JCK-1
....................ucuggaucuuguuugacaUc....................................................................................................................................................................................................   1      1    LT-1
....................ucuggaucuuguuugacagc....................................................................................................................................................................................................   41     0    LCK-3
....................ucuggaucuuguuugacagc....................................................................................................................................................................................................   127    0    LT-3
....................ucuggaucuuguuugacagc....................................................................................................................................................................................................   35     0    JCK-3
....................ucuggaucuuguuugacagc....................................................................................................................................................................................................   116    0    LT-2
....................ucuggCucuuguuugacagc....................................................................................................................................................................................................   1      1    LCK-1
....................ucuggaucuuguuugacagA....................................................................................................................................................................................................   1      1    JCK-3
....................ucuggaucuuguuugacagc....................................................................................................................................................................................................   55     0    JCK-2
....................ucuggCucuuguuugacagc....................................................................................................................................................................................................   2      1    LT-2
....................ucuggaucuuguuuNacagc....................................................................................................................................................................................................   1      1    LCK-1
....................uGuggaucuuguuugacagc....................................................................................................................................................................................................   1      1    LCK-1
....................ucuggaucuuguuuUacagc....................................................................................................................................................................................................   1      1    LCK-2
....................ucuggaucuuguuugacagA....................................................................................................................................................................................................   1      1    LT-3
....................ucuggaucuuguuugacagc....................................................................................................................................................................................................   75     0    LCK-2
....................ucuggaucuuguuugacagA....................................................................................................................................................................................................   1      1    JCK-1
....................ucuggaucuuguuugacGgca...................................................................................................................................................................................................   2      1    JCK-1
....................uNuggaucuuguuugacagca...................................................................................................................................................................................................   1      1    JCK-1
....................ucuggaucuuguuugacagcU...................................................................................................................................................................................................   7      1    LT-3
....................ucCggaucuuguuugacagca...................................................................................................................................................................................................   1      1    JCK-2
....................ucuggaucuugGuugacagca...................................................................................................................................................................................................   2      1    LT-1
....................ucuggaucuuguuugacagca...................................................................................................................................................................................................   195    0    LT-2
....................ucuggCucuuguuugacagca...................................................................................................................................................................................................   1      1    JCK-2
....................ucuggaucuuguuugCcagca...................................................................................................................................................................................................   2      1    LT-3
....................ucuggauUuuguuugacagca...................................................................................................................................................................................................   1      1    JCK-2
....................ucuggaucuuguuugacagcU...................................................................................................................................................................................................   3      1    JCK-3
....................ucuggaucuuguuugacagcU...................................................................................................................................................................................................   3      1    JCK-2
....................ucuggaucuuguuugGcagca...................................................................................................................................................................................................   1      1    LCK-3
....................ucuggaucuuguuugUcagca...................................................................................................................................................................................................   1      1    JCK-2
....................ucuggaucuuguuugacagUa...................................................................................................................................................................................................   1      1    JCK-2
....................uUuggaucuuguuugacagca...................................................................................................................................................................................................   12     1    JCK-2
....................ucuggaucuuguuugacagca...................................................................................................................................................................................................   9      0    LCK-1
....................ucuggaucuuguuugacagcU...................................................................................................................................................................................................   14     1    LT-2
....................ucuggaucuuguuugacagca...................................................................................................................................................................................................   17     0    LCK-2
....................ucuggaucuuguuugacagca...................................................................................................................................................................................................   145    0    LCK-3
....................ucuggaucCuguuugacagca...................................................................................................................................................................................................   1      1    JCK-2
....................ucuggaucuugGuugacagca...................................................................................................................................................................................................   1      1    JCK-1
....................ucuggaucuuguuugacagcU...................................................................................................................................................................................................   9      1    LCK-3
....................uUuggaucuuguuugacagca...................................................................................................................................................................................................   1      1    JCK-1
....................ucuggaucuuguuugacagca...................................................................................................................................................................................................   12     0    JT-3
....................ucuggaucuuguuugCcagca...................................................................................................................................................................................................   1      1    JCK-1
....................ucuggaucuuguuugacaUca...................................................................................................................................................................................................   3      1    JCK-3
....................ucuggaucuuguuugacagNa...................................................................................................................................................................................................   2      1    JCK-1
....................ucuggaucuuguuugacagca...................................................................................................................................................................................................   1097   0    JCK-2
....................ucuggaucuuguuugacaUca...................................................................................................................................................................................................   2      1    JCK-1
....................ucuggaucuuguuugacagca...................................................................................................................................................................................................   299    0    LT-1
....................ucuggaucuuguuugacaUca...................................................................................................................................................................................................   5      1    JCK-2
....................ucuggaucuuguuugacagcC...................................................................................................................................................................................................   1      1    LCK-3
....................ucuggaucuuguuugacagca...................................................................................................................................................................................................   267    0    LT-3
....................ucuggauAuuguuugacagca...................................................................................................................................................................................................   1      1    JCK-2
....................ucuggaucuCguuugacagca...................................................................................................................................................................................................   1      1    LT-3
....................ucuNgaucuuguuugacagca...................................................................................................................................................................................................   1      1    JCK-1
....................ucuggaucuuguuugacagca...................................................................................................................................................................................................   11     0    JT-2
....................ucCggaucuuguuugacagca...................................................................................................................................................................................................   1      1    JCK-1
....................ucuggaucuuguuugacagca...................................................................................................................................................................................................   832    0    JCK-3
....................ucuggaucuuguuugacagcU...................................................................................................................................................................................................   6      1    LT-1
....................ucuggaucuuguuugacagca...................................................................................................................................................................................................   29     0    JT-1
....................uUuggaucuuguuugacagca...................................................................................................................................................................................................   8      1    JCK-3
....................ucuggaucuuguuugacagca...................................................................................................................................................................................................   628    0    JCK-1
....................ucuggaucuuguuugUcagca...................................................................................................................................................................................................   1      1    JCK-3
....................ucuggaucuuguuugCcagca...................................................................................................................................................................................................   1      1    LT-1
....................ucuggaucuuguuugacagcU...................................................................................................................................................................................................   6      1    JCK-1
....................ucuggaucuuguuugGcagca...................................................................................................................................................................................................   1      1    LT-2
....................ucuggaucuuguuugacagcN...................................................................................................................................................................................................   1      1    JCK-1
....................ucuggaucuuguuugacagcac..................................................................................................................................................................................................   22     0    JCK-3
....................ucuggaucuuguuugacagcac..................................................................................................................................................................................................   27     0    LT-1
....................ucuggaucuuguuugacagcaU..................................................................................................................................................................................................   2      1    LT-2
....................ucuggaucuuguuugacagcac..................................................................................................................................................................................................   50     0    JCK-2
```

Mature **Star**

cccaccucucuuguuuaccuucuggaucuuguuugacagcacucccaacguuguuguuuggaugacauagaagugcugucgaacaugauccagaaggugcugucgaacaacauacaaaaagggguuuacuuuacguaccgcccaucacgaaaaaaaaaaaaaaauuguuuaauauuauuucaagauuuauuugaaugaaaauagaacagcuauuccuugguuucuuuaauuacuaaaacccuaugaucuua			
....................ucuggaucuuguuugacagcac....................	17	0	LT-2
....................ucuggaucuuguuugCcagcac....................	1	1	LT-2
....................ucuggaucuuguuugacagcac....................	24	0	LT-3
....................ucuggaucuuguuugacagcac....................	19	0	LCK-3
....................ucuggaucuuguuugacagcaU....................	1	1	LT-1
....................ucuggaucuuguuugacagcac....................	34	0	JCK-1
....................ucuggaucuuguuugacagcacu....................	5	0	JCK-2
....................ucuggaucuuguuugacagcacu....................	3	0	LT-1
....................ucuggaucuuguuugacagcaUu....................	1	1	LT-3
....................ucuggaucuuguuugacagcacu....................	2	0	JCK-3
....................ucuggaucuuguuugacagcacu....................	2	0	JCK-1
....................ucuggaucuuguuugacagcacuc....................	1	0	JCK-2
....................ucuggaucuuguuugacagcacuc....................	1	0	JCK-1
....................ucuggaucuuguuugacagcacuc....................	1	0	JCK-3
....................ucuggaucuuguuugacagcacuc....................	2	0	LT-3
.....................cuggaucuuguuugacagc....................	1	0	JCK-1
.....................cuggaucuuguuugacagc....................	13	0	LT-2
.....................cuggaucuuguuugacagc....................	7	0	JCK-2
.....................cuggaucuuguuugacagc....................	5	0	LCK-3
.....................cuggaucuuguuugacagc....................	1	0	LT-3
.....................cuggaucuuguuugacagc....................	2	0	JCK-3
.....................cuggaucuuguuugacagc....................	5	0	LT-1
.....................cuggaucuuguuugacagcU....................	1	1	JCK-3
.....................cuggaucuuguuugacagca....................	11	0	LT-2
.....................cuggaucuuguuugacagcU....................	4	1	JT-2
.....................cuggaucuuguuugacagca....................	15	0	LT-3
.....................cuggaucuuguuugacagcU....................	5	1	LT-2
.....................cuggaucuuguuugacagca....................	12	0	LCK-3
.....................cuggaucuuguuugacagca....................	61	0	JCK-2
.....................cuggaucuuguuugCcagca....................	1	1	JCK-1
.....................cuggaucuuguuugacagcU....................	1	1	JCK-2
.....................cuggaucuuguuugacagca....................	9	0	LT-1
.....................cuggaucuuguuugacagcU....................	1	1	LT-3
.....................cuggaucuuguuugacagca....................	58	0	JCK-1
.....................cuggaucuuguuugacagca....................	51	0	JCK-3
.....................cuggaucuuguuugacagcU....................	4	1	JT-3
.....................cuggaucuuguuugacagcac....................	1	0	LCK-3
.....................cuggaucuuguuugacagcac....................	3	0	LT-3
.....................cuggaucuuguuugacagcac....................	4	0	LT-2
.....................cuggaucuuguuugacagcacu....................	46	0	JCK-2
.....................cuggaucuuguuugacagcaUu....................	1	1	JCK-2
.....................cuggaucuuguuugacagcacu....................	13	0	LT-3
.....................cuggaucuuguuugacagcacu....................	4	0	LT-1
.....................cuggaucuuguuugacagcacu....................	22	0	JCK-3
.....................cuggaucuuguuugacagcacu....................	17	0	JCK-1
.....................cuggaucuuguuugacagcaUu....................	2	1	JCK-3
.....................cuggaucuuguuugacagcacu....................	7	0	LT-2
.....................cuggaucuuguuugacagcacu....................	15	0	LCK-3
.....................cuggaucuuguuugacagcacuc....................	3	0	JCK-3
.....................cuggaucuuguuugacagcacuc....................	1	0	JCK-2
.....................cuggaucuuguuugacagcacuc....................	1	0	JCK-1
......................uNgaucuuguuugacagc....................	1	1	JT-1
......................uggNucuuguuugacagc....................	1	1	JT-2
......................uggaucuuguuugacagc....................	5296	0	LT-2
......................uggaucuuguuuNacagc....................	3	1	LCK-1
......................uggaucuuguuugaNagc....................	11	1	LCK-1
......................uggaucuuguuugacCgc....................	2	1	JCK-2
......................uggCucuuguuugacagc....................	2	1	JCK-2
......................uggaucuuguuNgacagc....................	1	1	JCK-1
......................uggaucuuguuNgacagc....................	1	1	JCK-2
......................uggaucuuguuugacagU....................	13	1	LCK-3
......................uggaucuugCuugacagc....................	1	1	LT-1
......................uggauNuuguuugacagc....................	1	1	LCK-1
......................uggaucuuguuugacCgc....................	6	1	LT-2
......................uggaucuuguuugCcagc....................	2	1	LCK-2
......................uUgaucuuguuugacagc....................	1	1	LT-2
......................ugNaucuuguuugacagc....................	1	1	LCK-1
......................uggaucuuAuuugacagc....................	3	1	LT-2
......................ugAaucuuguuugacagc....................	1	1	JCK-2
......................uggCucuuguuugacagc....................	2	1	JT-3

```
                      Mature                                                   Star
cccaccucucuuguuuaccuucuggaucuuguuugacagcacucccaacguuguuguuuggaugacauagaagugcugucgaacaugauccagaaggugcugucgaacaacauacaaaagggguuuacuuuacguaccgcccaucacgaaaaaaaaaaaaaaauuguuuaauauuauuucaagauuuauuugaaugaaaauagaacagcuauuccuugguuucuuuaauuacuaaaacccuaugaucuua
......................uggaucuuguuugacaCc..................................................................................................................................................................................   2     1   JCK-3
......................uggaucuuguuugacagU..................................................................................................................................................................................   9     1   LT-1
......................uggaucuuguuugacagA..................................................................................................................................................................................   4     1   LCK-3
......................uggaucuuAuuugacagc..................................................................................................................................................................................   1     1   LT-3
......................uAgaucuuguuugacagc..................................................................................................................................................................................   4     1   LCK-3
......................uggaucuuguuuAacagc..................................................................................................................................................................................   1     1   LCK-3
......................ugUaucuuguuugacagc..................................................................................................................................................................................   1     1   LCK-3
......................uggCucuuguuugacagc..................................................................................................................................................................................   53    1   LT-2
......................uggaucuuguuuUacagc..................................................................................................................................................................................   1     1   JT-2
......................uggaucCuguuugacagc..................................................................................................................................................................................   1     1   LT-3
......................uggaucuuguuugacagc..................................................................................................................................................................................   2286  0   LT-1
......................uggaucuugCuugacagc..................................................................................................................................................................................   1     1   JCK-2
......................Cggaucuuguuugacagc..................................................................................................................................................................................   1     1   LT-3
......................uggaucuuguuugGcagc..................................................................................................................................................................................   2     1   LCK-1
......................uUgaucuuguuugacagc..................................................................................................................................................................................   1     1   LCK-3
......................uggaucuuguuugCcagc..................................................................................................................................................................................   3     1   JCK-1
......................uggaucuuguuugacGgc..................................................................................................................................................................................   1     1   LT-2
......................ugNaucuuguuugacagc..................................................................................................................................................................................   7     1   LT-2
......................uggaucuuguuugacagU..................................................................................................................................................................................   3     1   JCK-2
......................Cggaucuuguuugacagc..................................................................................................................................................................................   1     1   LT-2
......................uNgaucuuguuugacagc..................................................................................................................................................................................   1     1   JCK-1
......................uggaucuuguuugacagU..................................................................................................................................................................................   21    1   LT-2
......................uggaucuuguuugacagc..................................................................................................................................................................................   520   0   JT-1
......................uggaucuuguuugCcagc..................................................................................................................................................................................   1     1   JCK-3
......................uggCucuuguuugacagc..................................................................................................................................................................................   1     1   JCK-1
......................uggaucuuguuugacagG..................................................................................................................................................................................   1     1   JCK-1
......................uggaucuuguuugacagc..................................................................................................................................................................................   1810  0   JCK-2
......................uggaucuuguuNgacagc..................................................................................................................................................................................   1     1   JT-1
......................uggGucuuguuugacagc..................................................................................................................................................................................   1     1   LT-2
......................uggaucuuAuuugacagc..................................................................................................................................................................................   1     1   JCK-1
......................uggaucuuguuugacagA..................................................................................................................................................................................   3     1   JCK-1
......................uggaucuuguuugaGagc..................................................................................................................................................................................   4     1   LCK-1
......................uggNucuuguuugacagc..................................................................................................................................................................................   1     1   JCK-1
......................uggaucuuguuugCcagc..................................................................................................................................................................................   4     1   LT-3
......................uNgaucuuguuugacagc..................................................................................................................................................................................   1     1   LT-3
......................uggaucuuguuugacCgc..................................................................................................................................................................................   2     1   LCK-3
......................uNgaucuuguuugacagc..................................................................................................................................................................................   1     1   LCK-3
......................uAgaucuuguuugacagc..................................................................................................................................................................................   4     1   LT-3
......................ugAaucuuguuugacagc..................................................................................................................................................................................   1     1   LT-3
......................uggaucuuguuugacagA..................................................................................................................................................................................   7     1   LT-2
......................uggaucuuguuugCcagc..................................................................................................................................................................................   4     1   JCK-2
......................uggCucuuguuugacagc..................................................................................................................................................................................   5     1   LT-1
......................uggaucuuguuugacagc..................................................................................................................................................................................   1255  0   JCK-3
......................uggCucuuguuugacagc..................................................................................................................................................................................   2     1   JT-2
......................uggaucuuguuugacagc..................................................................................................................................................................................   1080  0   JCK-1
......................uAgaucuuguuugacagc..................................................................................................................................................................................   1     1   LT-1
......................uggGucuuguuugacagc..................................................................................................................................................................................   1     1   LCK-3
......................uNgaucuuguuugacagc..................................................................................................................................................................................   3     1   JCK-2
......................uggaucuuguuugacagc..................................................................................................................................................................................   1573  0   LCK-2
......................ugUaucuuguuugacagc..................................................................................................................................................................................   1     1   JCK-1
......................ugUaucuuguuugacagc..................................................................................................................................................................................   1     1   LT-2
......................uggaucuuguuugacagc..................................................................................................................................................................................   353   0   JT-2
......................uggaucuuguuugCcagc..................................................................................................................................................................................   6     1   LT-1
......................uggaucuugCuugacagc..................................................................................................................................................................................   1     1   JCK-1
......................uggaucuuguuugacagU..................................................................................................................................................................................   1     1   JCK-1
......................uggaucuuguuugacagU..................................................................................................................................................................................   12    1   LT-3
......................uggaucuuguuugacagA..................................................................................................................................................................................   1     1   LCK-1
......................ugAaucuuguuugacagc..................................................................................................................................................................................   3     1   LT-2
......................uggaucuuguGugacagc..................................................................................................................................................................................   1     1   LT-2
......................uggaucuuguuugacagA..................................................................................................................................................................................   1     1   JCK-3
......................uNgaucuuguuugacagc..................................................................................................................................................................................   2     1   LCK-1
......................uggCucuuguuugacagc..................................................................................................................................................................................   1     1   JCK-3
......................ugAaucuuguuugacagc..................................................................................................................................................................................   3     1   LCK-3
......................uggaucuuguuugCcagc..................................................................................................................................................................................   2     1   LCK-1
......................uggCucuuguuugacagc..................................................................................................................................................................................   7     1   LT-3
......................uggaucuCguuugacagc..................................................................................................................................................................................   1     1   LT-1
......................uggCucuuguuugacagc..................................................................................................................................................................................   2     1   LCK-1
......................ugAaucuuguuugacagc..................................................................................................................................................................................   1     1   LT-1
......................uggaucuuguuugacagA..................................................................................................................................................................................   6     1   LT-3
......................uggaucuuguuugacagc..................................................................................................................................................................................   2328  0   LT-3
```

```
                      Mature                                                 Star
cccaccucucuuguuuaccuucuggaucuuguuugacagcacucccaacguuguuguuuggaugacauagaagugcugucgaacaugauccagaaggugcugucgaacaacauacaaaaaggggguuuacuuuacguaccgcccaucacgaaaaaaaaaaaaaaauuguuuaauauuauuucaagauuuauuugaaugaaaauagaacagcuauuccuugguuucuuuaauuacuaaaacccuaugaucuua
......................uggaucuuguuugacagc      6351  0  LCK-3
......................uggaucuuguuugacCgc      1     1  JT-1
......................uggaucuuguuuAacagc      1     1  LCK-2
......................uggaucuAguuugacagc      1     1  LT-1
......................uAgaucuuguuugacagc      5     1  LT-2
......................uggaucuuguuugCcagc      15    1  LCK-3
......................uggaucuuAuuugacagc      1     1  LCK-2
......................uggCucuuguuugacagc      3     1  JT-1
......................uggaucuuguuugacCgc      2     1  LCK-2
......................uggaucuuguuugacagc      419   0  JT-3
......................uUgaucuuguuugacagc      1     1  JCK-2
......................uAgaucuuguuugacagc      1     1  LCK-2
......................uggaucuuguuugacagU      1     1  JCK-3
......................uggaucuuguuugacCgc      2     1  LT-1
......................ugAaucuuguuugacagc      2     1  LCK-1
......................uggCucuuguuugacagc      10    1  LCK-3
......................uggaucuuguuugacagc      1447  0  LCK-1
......................uCgaucuuguuugacagc      1     1  LT-1
......................uUgaucuuguuugacagc      1     1  LT-3
......................uggaucuuAuuugacagc      3     1  LT-1
......................uggCucuuguuugacagc      3     1  LCK-2
......................uNgaucuuguuugacagc      1     1  JCK-3
......................uggaucuuguCugacagc      2     1  JCK-2
......................uggaucuuguuugNcagc      6     1  LCK-1
......................uggaucuuguuugCcagc      24    1  LT-2
......................uggaucuuguuugNcagca     9     1  LCK-1
......................uggaucuuguuugGcagca     2     1  LCK-1
......................uggaucuuguuugacCgca     3     1  LCK-3
......................uNgaucuuguuugacagca     1     1  JCK-3
......................uggaucuuguuugacagca     1426  0  JT-1
......................ugUaucuuguuugacagca     1     1  LCK-2
......................uggaucuuguuugacagUa     12    1  JCK-1
......................Cggaucuuguuugacagca     1     1  LCK-3
......................uggaucuuguuuNacagca     2     1  LCK-1
......................ugAaucuuguuugacagca     1     1  JT-1
......................uggaucuuguuugacagcU     381   1  JT-3
......................uggaucuuguuugacagcG     6     1  JCK-1
......................uggaucCuguuugacagca     1     1  LT-2
......................uggaucuuguuugacagca     980   0  JT-2
......................uggaucuuguuGgacagca     9     1  LCK-3
......................uggCucuuguuugacagca     5     1  JT-1
......................uggaucuuguuugacagUa     10    1  JT-3
......................uggaucuuguuNgacagca     1     1  JT-2
......................uggaucuuguuugaAagca     1     1  LCK-3
......................uggaucuuguuugacagUa     11    1  JT-2
......................uggaucuugCuugacagca     1     1  JT-1
......................uggCucuuguuugacagca     2     1  LT-3
......................uggaucuuguuugacagcG     2     1  JCK-3
......................uggaucuuguuugacagca     3565  0  LCK-1
......................uggaucuuguuugacagcC     5     1  JCK-2
......................uAgaucuuguuugacagca     1     1  LT-3
......................uggaucuuguuugacagcU     1503  1  LCK-2
......................uggaucuuguuugCcagca     3     1  LCK-1
......................uggGucuuguuugacagca     1     1  JCK-1
......................uggaucuuguuugacagcU     718   1  JCK-1
......................uggaucuuguuugacagUa     3     1  LT-1
......................uggaucuuguuugacCgca     1     1  JCK-3
......................uggaucuuguuugCcagca     1     1  LT-1
......................uggCucuuguuugacagca     12    1  LT-2
......................uNgaucuuguuugacagca     2     1  JCK-2
......................uggCucuuguuugacagca     4     1  LCK-3
......................uggaucuuguuugacagcC     21    1  LT-3
......................uAgaucuuguuugacagca     3     1  LCK-3
......................uUgaucuuguuugacagca     1     1  LT-3
......................uggCucuuguuugacagca     3     1  JCK-3
......................uggaucuuguuugacagcC     7     1  LCK-1
......................uggaucuugNuugacagca     1     1  LCK-1
......................uggaucuuguuugCcagca     2     1  LCK-3
......................uggaucuuguuugacCgca     2     1  JCK-2
......................uggaucuuguuugGcagca     1     1  JT-2
```

Mature **Star**

cccaccucucuuguuuaccuucuggaucuuguuugacagcacucccaacguuguuguuuggaugacauagaagugcugucgaacaugauccagaaggugcugucgaacaacauacaaaagggguuuacuuuacguaccgcccaucacgaaaaaaaaaaaaaaauuguuuaauauuauuucaagauuuauuugaaugaaaauagaacagcuauuccuugguuucuuuaauuacuaaaacccuaugaucuua

uggaucuuguuugacagcU	4465	1	LCK-3
uggaucuuguuugacagcG	2	1	JCK-2
uggaucuuguuugacagcU	1354	1	LT-1
uggaucuuguuugacagcG	2	1	JT-2
uggaucuuguuugacagcU	835	1	JCK-3
uggaucuuguuugacagca	858	0	LT-1
uggaucuuguuugacagca	1294	0	JT-3
uggCucuuguuugacagca	4	1	JCK-2
uggaucuuAuuugacagca	1	1	LCK-3
ugNaucuuguuugacagca	9	1	LT-2
uNgaucuuguuugacagca	1	1	LCK-3
uggaucuuguuugacagcC	14	1	LT-1
uggaucuuguuugacCgca	2	1	LT-3
uggaucuuguuugacagcC	21	1	LCK-3
uggaucuuAuuugacagca	1	1	LCK-2
uggaucuuguuugacagUa	29	1	LT-2
uggCucuuguuugacagca	3	1	JT-2
uggCucuuguuugacagca	2	1	JCK-1
uggaucuuguuugacCgca	1	1	LT-2
uggaucuuguuugacagcU	735	1	JT-1
uggaucuuguuugaNagca	11	1	LCK-1
uggGucuuguuugacagca	1	1	LCK-2
uggaucuuguuugacagca	2333	0	LT-2
uggCucuuguuugacagca	7	1	LCK-2
uggaucuuguuugacagcU	2784	1	LT-2
uggaucuuguuugacagcC	8	1	LCK-2
uUgaucuuguuugacagca	5	1	JT-3
uggaucuuguuugacagUa	11	1	JCK-3
uggaucuuguuuGcagca	1	1	LT-2
uggaucuuguuugaGagca	4	1	LCK-1
uggaucuuguuugacagUa	18	1	LCK-3
uggCucuuguuugacagca	5	1	LCK-1
uggNucuuguuugacagca	1	1	LCK-1
uggaucuuguuugacagca	1154	0	JCK-1
uggaucuuguuugacagcU	279	1	JT-2
uggaucuuguuugCcagca	11	1	LT-2
uggaucuuguuugacagcG	15	1	LT-2
uggaucuuguuugacagAa	1	1	LT-3
uggaucuuguuugacCgca	1	1	JT-1
uggauUuuguuugacagca	1	1	LT-2
uAgaucuuguuugacagca	1	1	LT-2
uggaucuuguuugacagca	3633	0	LCK-2
uggaucuCguuugacagca	1	1	LCK-2
uggaucuuguuugacagca	3109	0	LCK-3
uggCucuuguuugacagca	2	1	LT-1
uggaucuuguuugacagUa	1	1	LCK-2
uggaucuuguuugacCgca	1	1	JCK-1
uNgaucuuguuugacagca	3	1	JT-1
uggaucuuguuugacagcU	1214	1	JCK-2
uggaucuuguuugCcagca	5	1	JCK-2
uggauAuuguuugacagca	1	1	JCK-3
uggaucuuguuugacagcU	2081	1	LT-3
uggaucuuguuugCcagca	6	1	LCK-2
ugAaucuuguuugacagca	1	1	LT-3
uggaucuuguuNgacagca	2	1	JT-1
uggaucuuguuugCcagca	2	1	LT-3
uNgaucuuguuugacagca	2	1	JCK-1
uggaucuuguuugacagcU	1372	1	LCK-1
uggaucuuguuugacagca	1105	0	JCK-3
uggaucGuguuugacagca	1	1	JT-3
uggaucuuguuNgacagca	5	1	JCK-1
uggaucuCguuugacagca	1	1	LT-2
uggaucuuguuugacagcC	2	1	JCK-1
uAgaucuuguuugacagca	1	1	LCK-2
uggaucuuguuugacagcC	20	1	LT-2
uggaucuuUuuugacagca	1	1	LCK-2
ugNaucuuguuugacagca	5	1	LCK-1
uggaCcuuguuugacagca	1	1	LT-2
uggaucuuguuugacagcG	11	1	LCK-3
uggaucuuguuugacagUa	24	1	JCK-2

Mature **Star**

cccaccucucuuguuuaccuucuggaucuuguuugacagcacucccaacguuguuguuuggaugacauagaagugcugucgaacaugauccagaaggugcugucgaacaacauacaaaaagggguuuacuuuacguaccgcccaucacgaaaaaaaaaaaaaauuguuuaauauuauuucaagauuuauuugaaugaaaauagaacagcuauuccuugguuucuuuaauuacuaaaacccuaugaucuua			
......................uggaucCuguuugacagca	1	1	LT-3
......................uggaucuuguuugacagca	1862	0	JCK-2
......................uggaucuuguuugacagca	1490	0	LT-3
......................uUgaucuuguuugacagca	5	1	JT-2
......................uggaucuuguuugacCgca	2	1	JT-3
......................uggaucuCguuugacagca	1	1	JT-1
......................uggaucCuguuugacagca	1	1	LCK-1
......................uggaucuCguuugacagca	1	1	LT-1
......................uggaucuuguuugaAagca	1	1	LCK-2
......................uggaucuuguuugacagcC	3	1	JT-1
......................uggCucuuguuugacagca	2	1	JT-3
......................uggaucuuguuugacCgca	2	1	LCK-1
......................uggaucuuguuugacagUa	11	1	LT-3
......................uggaucuuguuugCcagca	2	1	JCK-1
......................uggaucuuguuugacagcG	11	1	LT-3
......................uggaucuuguuugacagcC	6	1	JCK-3
......................uAgaucuuguuugacagca	1	1	JT-1
......................uggaucGuguuugacagca	1	1	LCK-2
......................uggauNuuguuugacagca	10	1	LCK-1
......................ugAaucuuguuugacagca	1	1	JCK-3
......................uggaucuuguuugacagcG	12	1	LT-1
......................uggaucuuguuuUacagca	1	1	LCK-3
......................uggauNuuguuugacagcac	1	1	LCK-1
......................uggaucuuguuugacagcaN	2	1	JCK-1
......................uggaucuuguuugacagcCc	1	1	JT-1
......................uggaucuuguuugacagcCc	1	1	LT-2
......................uggaucuuguuugacagcNc	1	1	JCK-1
......................uggaCcuuguuugacagcac	1	1	JCK-2
......................uggaucuuguCugacagcac	1	1	JCK-2
......................uggaucuugCuugacagcac	1	1	LT-2
......................uggaucuuguuugacGgcac	1	1	LT-2
......................uggaucuuguuugacagcac	666	0	LT-1
......................uggaucuuguuugacagcaA	3	1	JCK-1
......................uggaucuuguuugacagcaU	26	1	LT-2
......................uggaucuuguuugacagcac	170	0	JT-2
......................uggaucuuguuugacagcaU	8	1	JCK-1
......................uggaucuuguuugacagcac	715	0	LT-3
......................uggaucuuguuuAacagcac	1	1	LCK-2
......................ugAaucuuguuugacagcac	1	1	JCK-1
......................uggaucuuguuugacagUac	1	1	JCK-3
......................uAgaucuuguuugacagcac	1	1	LCK-1
......................uggaCcuuguuugacagcac	1	1	JT-1
......................uggaucuuguuugGcagcac	1	1	LT-3
......................uggaucuuguuugacagcaA	1	1	LCK-2
......................uggCucuuguuugacagcac	2	1	JCK-2
......................uggaucuuguuugacagcac	216	0	JT-1
......................uAgaucuuguuugacagcac	1	1	LT-1
......................uAgaucuuguuugacagcac	4	1	LT-2
......................uggaucuuguuugCcagcac	1	1	JCK-3
......................uggaucuuguuugacagcUc	26	1	LCK-1
......................uggaucuuguuugacagcCc	2	1	LT-1
......................uggaucuuguuugCcagcac	2	1	JT-1
......................uggaucuuguuugacagcaU	16	1	JT-1
......................uAgaucuuguuugacagcac	1	1	LCK-3
......................uggCucuuguuugacagcac	2	1	JT-3
......................uggaucuuguuugacagcUc	7	1	JCK-3
......................uggCucuuguuugacagcac	1	1	LCK-2
......................uNgaucuuguuugacagcac	1	1	LCK-3
......................ugNaucuuguuugacagcac	3	1	LT-2
......................uggaucuuguuugacagcac	525	0	LCK-2
......................uggaucuuguuugacagcac	982	0	LT-2
......................uggaucuuguuugacagcUc	51	1	LT-3
......................uggaucuuguuugacagcaA	3	1	LT-3
......................uggaucuuguuugacagcac	545	0	LCK-1
......................uggaucuuguuugacagcaU	66	1	LCK-3
......................uggaucuuguuugCcagcac	1	1	JT-2
......................uggaucuuguuugacagcaG	1	1	LT-1
......................uggCucuuguuugacagcac	5	1	LCK-3
......................uggaucuuguuugacagcac	586	0	JCK-1
......................uggaucuuguuugacagcac	249	0	JT-3

Mature **Star**

cccaccucucuuguuuaccuucuggaucuuguuugacagcacucccaacguuguuguuuggaugacauagaagugcugucgaacaugauccagaaggugcugucgaacaacauacaaaaaggggguuuacuuuacguaccgcccaucacgaaaaaaaaaaaaaaauuguuuaauauuauuucaagauuuauuugaaugaaaauagaacagcuauuccuugguuucuuuaauuacuaaaacccuaugaucuua

......................uggaucuuguuugacagcaA	1	1	JT-3
......................uggaucuuguuugacagcGc	1	1	LCK-3
......................uggaucuuguuugaNagcac	2	1	LCK-1
......................uggaucuuguuugGcagcac	1	1	LCK-1
......................uggaucuuguuugacagcUc	15	1	JCK-2
......................Aggaucuuguuugacagcac	1	1	LCK-3
......................uggaucuuguuugCcagcac	1	1	LCK-3
......................uggaucuuguuugacCgcac	3	1	LT-3
......................uggaucuuguuugCcagcac	1	1	LT-1
......................uggaucuuguuugacCgcac	2	1	JCK-2
......................uggaucuuguuugacagcac	899	0	JCK-2
......................uggaucuuguuugacagcUc	31	1	LCK-2
......................uggCucuuguuugacagcac	1	1	JCK-1
......................uggaucuuguuugacagcUc	63	1	LCK-3
......................uggaucuuguuugacagcUc	6	1	JCK-1
......................uNgaucuuguuugacagcac	1	1	JCK-1
......................uggCucuuguuugacagcac	1	1	LCK-1
......................uggaucuuguuugacagcUc	36	1	LT-1
......................uggaucuuUuuugacagcac	1	1	LT-3
......................uggaucuuguuugacagcaA	1	1	JCK-2
......................uggaucuuguuugCcagcac	1	1	LT-3
......................uggGucuuguuugacagcac	2	1	LT-2
......................uggCucuuguuugacagcac	2	1	LT-3
......................uggCucuuguuugacagcac	9	1	LT-2
......................uggaCcuuguuugacagcac	1	1	JCK-3
......................uggaucuuguuugCcagcac	3	1	JCK-2
......................uggaucuuguuNgacagcac	1	1	JT-2
......................uggaucuuguuugacagcaU	22	1	LT-1
......................uggaucuuguuugacagcac	1318	0	LCK-3
......................uggaucuuguuugacCgcac	1	1	LT-2
......................uggaucuuguuugCcagcac	4	1	LT-2
......................uggaucuuguuugNcagcac	2	1	LCK-1
......................uggCucuuguuugacagcac	1	1	LT-1
......................uggaucuuCuuugacagcac	1	1	LCK-3
......................uggaucuuguuugacagcUc	77	1	LT-2
......................uggaucuuguuugacagcaU	26	1	LT-3
......................uggaucuuguuugacagcaU	26	1	JCK-3
......................uggaucuuguuugCcagcac	1	1	JCK-1
......................uggaucuuguuugacagcac	687	0	JCK-3
......................uggaucuuguuugacagcaA	4	1	JCK-3
......................uggaucuuguuugacagcaU	46	1	JCK-2
......................ugAaucuuguuugacagcacu	8	1	JCK-3
......................uggGucuuguuugacagcacu	8	1	JCK-2
......................uggCucuuguuugacagcacu	65	1	LT-1
......................uggaucuuguuugacagcaNu	42	1	JCK-1
......................uggaucuugCuugacagcacu	6	1	JCK-2
......................uggaucuuguuugacagcacG	12	1	LT-3
......................ugUaucuuguuugacagcacu	3	1	LT-3
......................uggaucuuguuuCacagcacu	1	1	JCK-2
......................uggaucuuguuugNcagcacu	15	1	LCK-1
......................uggGucuuguuugacagcacu	8	1	JCK-1
......................ugUaucuuguuugacagcacu	1	1	JT-1
......................uggaucuuguuugacagAacu	1	1	JCK-2
......................uUgaucuuguuugacagcacu	2	1	LT-1
......................uggaucuuguGugacagcacu	15	1	JCK-1
......................uggaucuuguuugCcagcacu	2	1	JT-2
......................uggaucuuguuugacagcaUu	102	1	LCK-3
......................uggaucuuguuugacGgcacu	7	1	LT-2
......................uggaucuuguuugacagcacu	1709	0	JT-1
......................uggaucuuAuuugacagcacu	1	1	LCK-2
......................uggaucuuguuugacagcGcu	3	1	LT-2
......................uggaucuuguuugacCgcacu	29	1	JCK-2
......................uggaucuuguuGgacagcacu	1	1	JCK-3
......................uAgaucuuguuugacagcacu	15	1	JCK-3
......................uggaucuuguuuAacagcacu	2	1	LT-2
......................uggaucuuguuugacagcUcu	1	1	JCK-2
......................uggCucuuguuugacagcacu	9	1	LCK-2
......................ugUaucuuguuugacagcacu	2	1	JCK-1
......................uggaucuuguuugacagcacu	48441	0	JCK-3
......................uggaucuCguuugacagcacu	1	1	LCK-1

```
                      Mature                             Star
cccaccucucuuguuuaccuucuggaucuuguuugacagcacucccaacguuguuguuuggaugacauagaagugcugucgaacaugauccagaaggugcugucgaacaacauacaaaaagggguuuacuuuacguaccgcccaucacgaaaaaaaaaaaaaauuguuuaauauuauuucaagauuuauuugaaugaaaauagaacagcuauuccuugguuucuuuaauuacuaaaacccuaugaucuua
......................uggaucuuguuugacagcaUu...............................................................................................................................................................................   55      1    JT-1
......................uggaucuuAuuugacagcacu...............................................................................................................................................................................   20      1    JCK-2
......................uggaucuuguuugacagcacu...............................................................................................................................................................................   66730   0    JCK-2
......................uggaucuuguuuUacagcacu...............................................................................................................................................................................   6       1    JCK-1
......................uggaucuugGuugacagcacu...............................................................................................................................................................................   1       1    JCK-2
......................uggaucuuguuNgacagcacu...............................................................................................................................................................................   1       1    JCK-2
......................uggaucuuguuugCcagcacu...............................................................................................................................................................................   7       1    LCK-2
......................uggCucuuguuugacagcacu...............................................................................................................................................................................   39      1    LCK-3
......................uggaucuuguuugacagcCcu...............................................................................................................................................................................   29      1    LT-1
......................uggaucuuguuGgacagcacu...............................................................................................................................................................................   1       1    JCK-2
......................uggaucuuguuugacagcUcu...............................................................................................................................................................................   6       1    LT-2
......................uggaucuuguuugacagcCcu...............................................................................................................................................................................   4       1    LCK-2
......................Cggaucuuguuugacagcacu...............................................................................................................................................................................   7       1    JCK-3
......................uggaucuuguuugacagcCcu...............................................................................................................................................................................   74      1    JCK-2
......................uggaucuuguuugacagcacu...............................................................................................................................................................................   658     0    JT-2
......................uggCucuuguuugacagcacu...............................................................................................................................................................................   2       1    JT-2
......................uggaucCuguuugacagcacu...............................................................................................................................................................................   1       1    LCK-1
......................uggauGuuguuugacagcacu...............................................................................................................................................................................   1       1    JCK-3
......................uggaucuuguuugacagcCcu...............................................................................................................................................................................   31      1    LT-3
......................uggaucuuguuugaAagcacu...............................................................................................................................................................................   4       1    JCK-2
......................uggaucuuguCugacagcacu...............................................................................................................................................................................   1       1    LCK-2
......................uggaucCuguuugacagcacu...............................................................................................................................................................................   8       1    JCK-3
......................uggaucuuguuugacagcaAu...............................................................................................................................................................................   1       1    LT-1
......................uggaucuuguuugacagcUcu...............................................................................................................................................................................   1       1    LT-3
......................uAgaucuuguuugacagcacu...............................................................................................................................................................................   5       1    JCK-1
......................uggaucuuCuuugacagcacu...............................................................................................................................................................................   1       1    JCK-2
......................ugCaucuuguuugacagcacu...............................................................................................................................................................................   1       1    JCK-3
......................uggaucuuUuuugacagcacu...............................................................................................................................................................................   2       1    LT-1
......................uggCucuuguuugacagcacu...............................................................................................................................................................................   118     1    JCK-3
......................ugUaucuuguuugacagcacu...............................................................................................................................................................................   1       1    LCK-2
......................uggaCcuuguuugacagcacu...............................................................................................................................................................................   1       1    LCK-2
......................uggaucuuguuugacagcacG...............................................................................................................................................................................   3       1    LT-2
......................uggaucuuguuuAacagcacu...............................................................................................................................................................................   1       1    LCK-3
......................Cggaucuuguuugacagcacu...............................................................................................................................................................................   11      1    JCK-2
......................uggCucuuguuugacagcacu...............................................................................................................................................................................   95      1    LT-3
......................uggaucuuguuugacagcacN...............................................................................................................................................................................   22      1    JCK-1
......................uggauUuuguuugacagcacu...............................................................................................................................................................................   2       1    LT-3
......................uNgaucuuguuugacagcacu...............................................................................................................................................................................   66      1    JCK-1
......................uggaucuuguuugacagcaUu...............................................................................................................................................................................   36      1    LCK-1
......................uggaucuuguuugacagcCcu...............................................................................................................................................................................   17      1    JCK-3
......................uggNucuuguuugacagcacu...............................................................................................................................................................................   3       1    JCK-3
......................uggaucuuguuugGcagcacu...............................................................................................................................................................................   3       1    JCK-1
......................uggaucuCguuugacagcacu...............................................................................................................................................................................   5       1    JCK-1
......................uggaCcuuguuugacagcacu...............................................................................................................................................................................   14      1    JCK-2
......................uggaucuuAuuugacagcacu...............................................................................................................................................................................   6       1    LT-1
......................uggaucuuguGugacagcacu...............................................................................................................................................................................   2       1    LT-2
......................uggauAuuguuugacagcacu...............................................................................................................................................................................   1       1    JCK-3
......................uggGucuuguuugacagcacu...............................................................................................................................................................................   5       1    JCK-3
......................uggaucuuguuugacagcacu...............................................................................................................................................................................   4848    0    LCK-1
......................uUgaucuuguuugacagcacu...............................................................................................................................................................................   2       1    LCK-3
......................uggaucuuguuuCacagcacu...............................................................................................................................................................................   1       1    JCK-3
......................uggaucuuguuuAacagcacu...............................................................................................................................................................................   9       1    JCK-2
......................uUgaucuuguuugacagcacu...............................................................................................................................................................................   4       1    JCK-2
......................uggaCcuuguuugacagcacu...............................................................................................................................................................................   13      1    JCK-3
......................uggNucuuguuugacagcacu...............................................................................................................................................................................   22      1    JCK-1
......................uggaCcuuguuugacagcacu...............................................................................................................................................................................   2       1    LT-2
......................Cggaucuuguuugacagcacu...............................................................................................................................................................................   3       1    LT-1
......................uggaucuuguuuAacagcacu...............................................................................................................................................................................   3       1    LT-1
......................uggaucuuguuugacaCcacu...............................................................................................................................................................................   5       1    JCK-3
......................uggaucuuguuugacGgcacu...............................................................................................................................................................................   3       1    LT-3
......................uggaucuugCuugacagcacu...............................................................................................................................................................................   2       1    JCK-1
......................uggaucuuguuugacagcaAu...............................................................................................................................................................................   3       1    JCK-1
......................uggaucuuguuGgacagcacu...............................................................................................................................................................................   10      1    JCK-1
......................uggaucuuguuugacagcaUu...............................................................................................................................................................................   21      1    JT-3
......................uggCucuuguuugacagcacu...............................................................................................................................................................................   152     1    JCK-2
......................Cggaucuuguuugacagcacu...............................................................................................................................................................................   9       1    LT-3
......................uggaucuuguuugacCgcacu...............................................................................................................................................................................   19      1    JCK-1
......................uggaucuuguuugacagcaUu...............................................................................................................................................................................   20      1    LT-1
......................uggaucuuguuuUacagcacu...............................................................................................................................................................................   6       1    JCK-2
......................uggaucuuguuugacCgcacu...............................................................................................................................................................................   26      1    LT-2
```

Mature (red) … **Star** (purple)

cccaccucucuuguuuaccuuc**uggaucuuguuugacagcacu**cccaacguuguuguuuggaugacauagaag**ugcugucgaacaugauccaga**aggugcugucgaacaacauacaaaaagggguuuacuuuacguaccgcccaucacgaaaaaaaaaaaaaaauuguuuaauauuauuucaagauuuauuugaaugaaaauagaacagcuauuccuugguuucuuuaauuacuaaaacccuaugaucuua

......................uggaucuuguuugacagUacu	34	1	JCK-2
......................uggaucuuguuugacagcaUu	2361	1	JCK-2
......................uggaucuuguuugacagAacu	1	1	JCK-1
......................uggaucuuguuuUacagcacu	3	1	LT-3
......................uUgaucuuguuugacagcacu	10	1	JCK-3
......................ugUaucuuguuugacagcacu	2	1	LT-2
......................uggaucuuguuugacagUacu	13	1	JCK-3
......................ugAaucuuguuugacagcacu	4	1	LCK-3
......................uggaucuugCuugacagcacu	4	1	LT-2
......................uggaucuuguuugacagcCcu	34	1	LT-2
......................ugNaucuuguuugacagcacu	65	1	LT-2
......................uggaucuuUuuugacagcacu	3	1	LT-2
......................uAgaucuuguuugacagcacu	8	1	JCK-2
......................uggaucuuguCugacagcacu	6	1	JCK-1
......................uAgaucuuguuugacagcacu	13	1	LCK-3
......................uggGucuuguuugacagcacu	1	1	JT-1
......................uggNucuuguuugacagcacu	1	1	JCK-2
......................uggNucuuguuugacagcacu	1	1	LCK-1
......................uggaucuuguuugacGgcacu	2	1	LCK-3
......................uggaucuCguuugacagcacu	3	1	LT-3
......................uggauNuuguuugacagcacu	1	1	JCK-2
......................uggaucuuguuugacCgcacu	12	1	LT-1
......................uggaucuuNuuugacagcacu	1	1	JCK-3
......................uggaucuuguuugacagcaAu	1	1	LCK-3
......................uggaucuuguuuUacagcacu	2	1	JCK-3
......................ugAaucuuguuugacagcacu	3	1	JCK-1
......................uggaucuugCuugacagcacu	6	1	JCK-3
......................uggaucCuguuugacagcacu	2	1	LT-3
......................uggaucCuguuugacagcacu	12	1	JCK-2
......................uggaucuuguuugCcagcacu	5	1	JT-1
......................uggaucuuguuugacagcGcu	2	1	JCK-2
......................uggaucuuguuugacagcacu	23834	0	LT-1
......................uggaucuuguuuAacagcacu	7	1	JCK-3
......................uggaucuugCuugacagcacu	2	1	LT-1
......................uggaucuuguuugacagcaAu	5	1	JT-2
......................uggCucuuguuugacagcacu	92	1	JCK-1
......................uggaucuuUuuugacagcacu	1	1	LT-3
......................uggaucuuguuugCcagcacu	86	1	JCK-1
......................Gggaucuuguuugacagcacu	1	1	JCK-2
......................ugNaucuuguuugacagcacu	1	1	LCK-1
......................uggCucuuguuugacagcacu	7	1	JT-1
......................uggaucuuguuugacagcaUu	22	1	JT-2
......................uggaucuuguuugaGagcacu	1	1	JT-1
......................uggaucuuguuugacagcaAu	4	1	LT-2
......................uggaucuuguuugacagcCcu	53	1	JCK-1
......................uggaucuuguuugacagcacG	10	1	JCK-1
......................uNgaucuuguuugacagcacu	104	1	JCK-2
......................uggaucuuguuuAacagcacu	6	1	JCK-1
......................uggaucuuAuuugacagcacu	3	1	LT-3
......................uggauUuuguuugacagcacu	3	1	JCK-1
......................uggaucuuguuugacagUacu	1	1	JT-2
......................uggaucuuguuugaGagcacu	8	1	LCK-1
......................uggaucuugCuugacagcacu	4	1	LCK-3
......................uggaucuuguuugacaCcacu	5	1	JCK-1
......................uggaucuuguuugCcagcacu	61	1	LT-1
......................uggaucuuguuuAacagcacu	1	1	JT-1
......................uggaucuuguuugacagcNcu	22	1	JCK-1
......................ugCaucuuguuugacagcacu	2	1	LT-2
......................uggaucuuguuugacagcacG	2	1	LCK-1
......................uUgaucuuguuugacagcacu	7	1	LT-2
......................uggaucuuguuugCcagcacu	67	1	LT-3
......................uggaucuuUuuugacagcacu	3	1	JCK-1
......................uggaucuuguuugacagGacu	2	1	JCK-2
......................uggaucuuguuugacagcacu	20839	0	LT-2
......................uggaucuuguuAgacagcacu	1	1	LCK-3
......................uAgaucuuguuugacagcacu	22	1	LT-3
......................uggaucuuguuugacagcCcu	16	1	LCK-3
......................uggaCcuuguuugacagcacu	4	1	LT-3
......................uggaucuuguuugacGgcacu	1	1	JT-3
......................uggaucuuguuugacagcaAu	3	1	LT-3

```
                      Mature                           Star
cccaccucucuuguuuaccuucuggaucuuguuugacagcacucccaacguuguuguuuggaugacauagaagugcugucgaacaugauccagaaggugcugucgaacaacauacaaaaggguuuacuuuacguaccgcccaucacgaaaaaaaaaaaaaaauuguuuaauauuauuucaagauuuauuugaaugaaaauagaacagcuauuccuugguuucuuuaauuacuaaaacccuaugaucuua
......................uggaucuuguuugCcagcacu..........................................   36      1   LCK-3
......................uggaucuuguuugacagcacu..........................................   20263   0   LCK-3
......................uggaucuuguCugacagcacu..........................................   7       1   JCK-3
......................uggaucuuguuugaAagcacu..........................................   3       1   LT-1
......................uggaucuuguuuNacagcacu..........................................   1       1   LCK-1
......................uggaucuuguuugacagcacG..........................................   10      1   LT-1
......................Cggaucuuguuugacagcacu..........................................   4       1   LT-2
......................uggaucuuguuugacagcCcu..........................................   4       1   LCK-1
......................uggaucuuAuuugacagcacu..........................................   4       1   LCK-3
......................uggaucuuUuuugacagcacu..........................................   6       1   JCK-3
......................ugUaucuuguuugacagcacu..........................................   4       1   JCK-3
......................uggauNuuguuugacagcacu..........................................   3       1   LCK-1
......................uggaucuuguuugCcagcacu..........................................   159     1   JCK-2
......................uggaucuuguuugGcagcacu..........................................   3       1   JCK-3
......................uggaucuuguuugacagcaUu..........................................   64      1   LT-3
......................ugAaucuuguuugacagcacu..........................................   10      1   LT-2
......................uggaucuuguuugacagcaGu..........................................   2       1   JCK-1
......................Cggaucuuguuugacagcacu..........................................   1       1   JT-1
......................uggaCcuuguuugacagcacu..........................................   3       1   LT-1
......................uNgaucuuguuugacagcacu..........................................   55      1   JCK-3
......................uggaucuuguuugacGgcacu..........................................   10      1   JCK-2
......................uggaucuuguuugacagcaAu..........................................   10      1   JCK-2
......................uggauUuuguuugacagcacu..........................................   4       1   JCK-3
......................ugAaucuuguuugacagcacu..........................................   19      1   JCK-2
......................uggaucuCguuugacagcacu..........................................   10      1   JCK-2
......................uggaucuuguuugaNagcacu..........................................   17      1   LCK-1
......................uggaucuuguuugacagUacu..........................................   5       1   LT-3
......................uggaucuCguuugacagcacu..........................................   3       1   LCK-3
......................uggaucuuguuugacagcacu..........................................   38814   0   JCK-1
......................uggaucuugAuugacagcacu..........................................   1       1   JCK-2
......................uggaucuuUuuugacagcacu..........................................   4       1   JCK-2
......................uggaucuCguuugacagcacu..........................................   1       1   LT-1
......................uggaucuuguuugacagcaAu..........................................   1       1   LCK-2
......................uUgaucuuguuugacagcacu..........................................   1       1   JT-3
......................uggaucuuguCugacagcacu..........................................   1       1   LT-3
......................ugUaucuuguuugacagcacu..........................................   1       1   JCK-2
......................uggaucuuguuugacagNacu..........................................   3       1   JCK-3
......................uggaucuuguuGgacagcacu..........................................   1       1   LT-3
......................uggaucuuguuugacGgcacu..........................................   9       1   JCK-3
......................uggaucuuguuugGcagcacu..........................................   3       1   JCK-2
......................uggaucuuguuugacCgcacu..........................................   3       1   JT-1
......................uNgaucuuguuugacagcacu..........................................   2       1   LT-3
......................uggaucuuguuuCacagcacu..........................................   2       1   JCK-1
......................uggaucuuCuuugacagcacu..........................................   1       1   JCK-1
......................uggaucuuguuugacCgcacu..........................................   5       1   LCK-2
......................uggaucuuguuugacagcacG..........................................   13      1   JCK-3
......................uggaucuuguuuAacagcacu..........................................   1       1   LT-3
......................uggaucuuguCugacagcacu..........................................   3       1   LT-1
......................uggaucuuguuugCcagcacu..........................................   105     1   JCK-3
......................ugAaucuuguuugacagcacu..........................................   11      1   LT-3
......................uggaucuuAuuugacagcacu..........................................   6       1   JCK-1
......................uggaucuuguuAgacagcacu..........................................   1       1   JCK-2
......................uggaucuugGuugacagcacu..........................................   1       1   JCK-1
......................uAgaucuuguuugacagcacu..........................................   14      1   LT-1
......................uggaucuuguuugacagcacG..........................................   7       1   LCK-3
......................uggaucuuguuugacagGacu..........................................   24      1   JCK-1
......................uggCucuuguuugacagcacu..........................................   200     1   LT-2
......................uggaucuugCuugacagcacu..........................................   1       1   LT-3
......................uggaucuuguuugacCgcacu..........................................   28      1   JCK-3
......................uggaucuuguuugacaCcacu..........................................   1       1   LT-3
......................uggaucuuguuugacGgcacu..........................................   7       1   JCK-1
......................uggaucuuguuugacagUacu..........................................   11      1   LT-2
......................uggCucuuguuugacagcacu..........................................   1       1   JT-3
......................uggaucuuguuNgacagcacu..........................................   1       1   JT-1
......................uggaucuuguuugUcagcacu..........................................   2       1   JCK-2
......................uggaucuuguuugacaCcacu..........................................   3       1   JCK-2
......................uggaucuuguuugacagcacu..........................................   979     0   JT-3
......................uggaucuuguuuAacagcacu..........................................   1       1   LCK-1
......................uggaucCuguuugacagcacu..........................................   2       1   LT-2
......................uggaucGuguuugacagcacu..........................................   2       1   JCK-1
```

Mature Star

cccaccucucuuguuuaccuucuggaucuuguuugacagcacucccaacguuguuguuuggaugacauagaagugcugucgaacaugauccagaaggugcugucgaacaacauacaaaaaggguuuacuuuacguaccgcccaucacgaaaaaaaaaaaaaaauuguuuaauauuauuucaagauuuauuugaaugaaaauagaacagcuauuccuugguuucuuuaauuacuaaaacccuaugaucuua

uggaucuuguuugacagcacu	5029	0	LCK-2
uggaGcuuguuugacagcacu	2	1	JCK-3
uggaucuuguuugacCgcacu	11	1	LT-3
uggaucuuguuugacGgcacu	2	1	LT-1
uggaucuuguuugacCgcacu	7	1	LCK-3
uCgaucuuguuugacagcacu	1	1	JCK-3
uNgaucuuguuugacagcacu	1	1	LCK-2
uggaucuuAuuugacagcacu	9	1	LT-2
uggaucuuCuuugacagcacu	1	1	JCK-3
uggaucuuguuugCcagcacu	80	1	LT-2
uggaCcuuguuugacagcacu	4	1	JCK-1
ugAaucuuguuugacagcacu	3	1	LCK-2
uAgaucuuguuugacagcacu	30	1	LT-2
uggaucuuguuugacagcGcu	1	1	JT-3
ugAaucuuguuugacagcacu	2	1	LCK-1
uggaucCuguuugacagcacu	10	1	JCK-1
uggaucuuguuugacagcaUu	1426	1	JCK-1
Cggaucuuguuugacagcacu	6	1	JCK-1
uggaucuuUuuugacagcacu	1	1	LCK-3
uggaucuuguuugaAagcacu	2	1	JCK-1
uggaucuuguuugacagcGcu	2	1	JCK-1
uNgaucuuguuugacagcacu	4	1	JT-1
uggaucuuguuuUacagcacu	2	1	LT-2
Cggaucuuguuugacagcacu	1	1	JT-3
uggaucuuAuuugacagcacu	1	1	LCK-1
uggaucuugGuugacagcacu	1	1	JCK-3
uggCucuuguuugacagcacu	8	1	LCK-1
uggaucuuguuugacagcacG	1	1	JT-2
uggauUuuguuugacagcacu	1	1	LT-2
uggaCcuuguuugacagcacu	1	1	LCK-3
uggaucuuguuugCcagcacu	1	1	JT-3
uggauAuuguuugacagcacu	2	1	JCK-2
uggaucuuguuugGcagcacu	2	1	LT-1
uggaucuuguuugacagAacu	3	1	LT-1
uggaucuuguuugCcagcacu	5	1	LCK-1
uggaucGuguuugacagcacu	1	1	LT-1
uggaucuuguuugacagGacu	2	1	JCK-3
uggaucuuguuugacagcNcu	1	1	LCK-1
uggGucuuguuugacagcacu	3	1	LT-3
uggauUuuguuugacagcacu	11	1	JCK-2
uggaucuuAuuugacagcacu	4	1	JCK-3
uggaucuuguuugacagcaUu	68	1	LT-2
uCgaucuuguuugacagcacu	1	1	LCK-3
uggaucuuguuugacagcCcu	2	1	JT-1
uggaucuuguuNgacagcacu	121	1	JCK-1
ugAaucuuguuugacagcacu	6	1	LT-1
uNgaucuuguuugacagcacu	1	1	LCK-1
uggaucuuguuugacagcacG	16	1	JCK-2
uggaucuCguuugacagcacu	4	1	JCK-3
uggaucuuguuugGcagcacu	1	1	LT-2
uggaucuuguuugaAagcacu	5	1	JCK-3
uNgaucuuguuugacagcacu	3	1	LT-1
uggaucuuguuugacagUacu	18	1	JCK-1
uggaucuuguuugacagcaAu	3	1	JCK-3
uggaucCuguuugacagcacu	4	1	LT-1
uggaucuuguCugacagcacu	1	1	LT-2
uggaucuuguuugacaCcacu	1	1	JT-1
uggaucuuguuuUacagcacu	4	1	LT-1
ugCaucuuguuugacagcacu	1	1	LCK-3
uggaucuGguuugacagcacu	1	1	JCK-2
uggauUuuguuugacagcacu	1	1	LCK-3
uggaucuuguuugacagcaAu	4	1	JT-3
uggaucuuguuugacCgcacu	3	1	LCK-1
uAgaucuuguuugacagcacu	4	1	LCK-2
uggaucuuguuugacagcaUu	1732	1	JCK-3
uggaucuuguuugGcagcacu	1	1	LT-3
uggaucuuguCugacagcacu	6	1	JCK-2
uggaucuuguuugacagcaUu	37	1	LCK-2
uggaucuuguuugGcagcacu	5	1	LCK-1
uggaucuuguuugacagcUcu	5	1	LT-1

Mature **Star**

Sequence			
cccaccucucuuguuuaccuuc**uggaucuuguuugacagcacu**cccaacguuguuguuuggaugacauagaag**ugcugucgaacaugauccaga**aggugcugucgaacaacauacaaaagggguuuacuuuacguaccgcccaucacgaaaaaaaaaaaaauuguuuaauauuauuucaagauuuauuugaaugaaaauagaacagcuauuccuugguuucuuuaauuacuaaaacccuaugaucuua			
......................uggaucuuguuAgacagcacu	1	1	JCK-1
......................uggaucuuguCugacagcacu	5	1	JT-2
......................uggaucuuguuugacagcacu	27651	0	LT-3
......................uggaucuuguuugacagcacNc	3	1	JCK-1
......................uggaucuugCuugacagcacuc	1	1	JCK-3
......................uggaucuuguuugacagcaUuc	3	1	LT-2
......................uggaucuuguuugacagcacuA	11	1	JT-1
......................uUgaucuuguuugacagcacuc	1	1	LT-2
......................uggaucuuguuugCcagcacuc	7	1	LT-1
......................uggaucuCguuugacagcacuc	2	1	JCK-3
......................uggaucuuguuugaAagcacuc	4	1	JCK-2
......................uggaucuuAuuugacagcacuc	1	1	LT-2
......................uUgaucuuguuugacagcacuc	1	1	JCK-3
......................uggaucuuguuugCcagcacuc	24	1	JCK-1
......................uggaucuuguuugacagcaAuc	1	1	JCK-2
......................uggaucuuguuugacagcacGc	1	1	JCK-1
......................Cggaucuuguuugacagcacuc	1	1	LCK-3
......................uggauUuuguuugacagcacuc	7	1	JCK-2
......................uggauAuuguuugacagcacuc	1	1	JCK-1
......................uggaucuuNuuugacagcacuc	1	1	JCK-3
......................uggCucuuguuugacagcacuc	4	1	LT-2
......................uggaucuuguuugacagcUcuc	1	1	JCK-1
......................uggaucuuguuugGcagcacuc	1	1	JCK-1
......................uggaucuuguuugacagcacuU	47	1	LT-1
......................uggaucuuguuugacagcacuU	97	1	JCK-2
......................uggaucuuguuugacagNacuc	1	1	JCK-3
......................uggaucuuguuugCcagcacuc	2	1	LT-3
......................uggaucuuguuugaGagcacuc	4	1	JCK-2
......................uggaucuuguuugacagcUcuc	2	1	JCK-2
......................uggaucuuguuugacCgcacuc	13	1	JCK-2
......................uggaucuuguuugacagcGcuc	3	1	JCK-2
......................uggaucuugGuugacagcacuc	3	1	JCK-1
......................ugNaucuuguuugacagcacuc	2	1	LT-2
......................uAgaucuuguuugacagcacuc	3	1	JCK-1
......................uggaucuuguuugacCgcacuc	2	1	LCK-3
......................uggaucuugGuugacagcacuc	3	1	JCK-3
......................uggaucuugCuugacagcacuc	1	1	JCK-2
......................uggaucuuguuuUacagcacuc	1	1	JCK-3
......................uggaucuuguuugCcagcacuc	19	1	JCK-3
......................uggaucuuAuuugacagcacuc	4	1	JCK-3
......................uggaucuuguuugacagcacuc	152	0	JT-3
......................uggaucuuguuugacagcacuG	1	1	LT-2
......................uAgaucuuguuugacagcacuc	2	1	LT-1
......................uggaucuuguuugacagGacuc	7	1	JCK-1
......................uggaucuuguuugCcagcacuc	2	1	LT-2
......................uggaucuuguuugacCgcacuc	1	1	LT-2
......................uggaucuuguuugacagcCcuc	1	1	LCK-1
......................uggaucuuguuugacagcacuN	9	1	JCK-1
......................uggaucuuguuugacagcacuN	4	1	LT-2
......................uggaucuuguCugacagcacuc	1	1	JCK-1
......................uggGucuuguuugacagcacuc	1	1	JCK-3
......................uggaucuuguuugacagcacuG	11	1	JCK-1
......................uggaucuuguuugacagcacuA	12	1	LCK-3
......................uggaucuuguuugacagcacuc	627	0	LT-2
......................uggaucuuguuugacagcacuA	32	1	LT-1
......................uggaucuuguuugacagcacuc	17179	0	JCK-2
......................uggaucuuguuugacagcacuA	38	1	JCK-1
......................uggaucuuguuugacagcaUuc	8	1	JCK-3
......................uggaucuuguuugacagcacuc	9941	0	JCK-1
......................uNgaucuuguuugacagcacuc	26	1	JCK-1
......................uggCucuuguuugacagcacuc	36	1	JCK-1
......................uggaucuuguuugCcagcacuc	44	1	JCK-2
......................uggaGcuuguuugacagcacuc	1	1	JCK-3
......................uggaucuuguuugacagcaUuc	7	1	JCK-1
......................uggaucuuguuugNcagcacuc	1	1	LCK-1
......................Cggaucuuguuugacagcacuc	2	1	JCK-1
......................uggaucuuguuGgacagcacuc	3	1	JCK-1
......................uggaucuuguuuUacagcacuc	1	1	JCK-1
......................uggaucuuguuugGcagcacuc	3	1	JCK-2
......................uggaucuCguuugacagcacuc	3	1	JCK-2

```
                      Mature                                                                  Star
cccaccucucuuguuuaccuucuggaucuuguuugacagcacucccaacguuguuguuuggaugacauagaagugcugucgaacaugauccagaaggugcugucgaacaacauacaaaaaggguuuacuuuacguaccgcccaucacgaaaaaaaaaaaaaaauuguuuaauauuauuucaagauuuauuugaaugaaaauagaacagcuauuccuugguuucuuuaauuacuaaaacccuaugaucuua
......................ugAaucuuguuugacagcacuc..............................................................................................................................................................................................................   2     1   LT-3
......................uggaucuuUuuugacagcacuc..............................................................................................................................................................................................................   2     1   JCK-2
......................uggaucuuguuugacagcacuU..............................................................................................................................................................................................................   68    1   JCK-1
......................uggaucuugGuugacagcacuc..............................................................................................................................................................................................................   3     1   JCK-2
......................uggaucuuguuugacagcCcuc..............................................................................................................................................................................................................   7     1   JCK-1
......................uggaucuuguuugCcagcacuc..............................................................................................................................................................................................................   1     1   JT-2
......................uggaucuuguuugacagcacuU..............................................................................................................................................................................................................   85    1   JCK-3
......................ugAaucuuguuugacagcacuc..............................................................................................................................................................................................................   2     1   JCK-1
......................uggGucuuguuugacagcacuc..............................................................................................................................................................................................................   1     1   JCK-1
......................uggauUuuguuugacagcacuc..............................................................................................................................................................................................................   1     1   JCK-3
......................uggaucuuguuugacagcacuU..............................................................................................................................................................................................................   1     1   JT-2
......................uggaucuuguuugacagcacuA..............................................................................................................................................................................................................   41    1   JCK-3
......................uggaucuuguGugacagcacuc..............................................................................................................................................................................................................   5     1   JCK-1
......................uggaucuuguuugacagcacuU..............................................................................................................................................................................................................   4     1   JT-3
......................uggaucuuguuNgacagcacuc..............................................................................................................................................................................................................   29    1   JCK-1
......................uAgaucuuguuugacagcacuc..............................................................................................................................................................................................................   1     1   LT-2
......................uggauUuuguuugacagcacuc..............................................................................................................................................................................................................   5     1   JCK-1
......................uggaucuuguuugacCgcacuc..............................................................................................................................................................................................................   3     1   JCK-3
......................uggaucuuguuugCcagcacuc..............................................................................................................................................................................................................   2     1   JT-1
......................uggaucuuguuugacagcCcuc..............................................................................................................................................................................................................   1     1   JT-3
......................uggaucuuguuugacagcaUuc..............................................................................................................................................................................................................   5     1   JCK-2
......................ugUaucuuguuugacagcacuc..............................................................................................................................................................................................................   3     1   JCK-3
......................uggaCcuuguuugacagcacuc..............................................................................................................................................................................................................   6     1   JCK-1
......................uggCucuuguuugacagcacuc..............................................................................................................................................................................................................   2     1   LCK-1
......................uggaucuuguuugacagcacuc..............................................................................................................................................................................................................   1069  0   LT-3
......................uggaucuuguuugacagcacuG..............................................................................................................................................................................................................   4     1   JCK-2
......................uggaucuuguuuAacagcacuc..............................................................................................................................................................................................................   1     1   JCK-3
......................ugAaucuuguuugacagcacuc..............................................................................................................................................................................................................   2     1   JCK-3
......................uggCucuuguuugacagcacuc..............................................................................................................................................................................................................   1     1   JT-1
......................uNgaucuuguuugacagcacuc..............................................................................................................................................................................................................   15    1   JCK-3
......................uUgaucuuguuugacagcacuc..............................................................................................................................................................................................................   1     1   JCK-1
......................uggCucuuguuugacagcacuc..............................................................................................................................................................................................................   1     1   JT-2
......................uggaucuuguuugacagcacuA..............................................................................................................................................................................................................   34    1   LT-3
......................uggaucCuguuugacagcacuc..............................................................................................................................................................................................................   2     1   JCK-2
......................uggaucuuguuugacCgcacuc..............................................................................................................................................................................................................   1     1   LT-1
......................uggaucCuguuugacagcacuc..............................................................................................................................................................................................................   1     1   JCK-1
......................uggaucuuguuugacagcacuc..............................................................................................................................................................................................................   70    0   LCK-2
......................uggaucuuguuugacagcacuA..............................................................................................................................................................................................................   33    1   LT-2
......................uggaucuuguuugacagUacuc..............................................................................................................................................................................................................   5     1   JCK-2
......................uggaucuuguuugacagcacGc..............................................................................................................................................................................................................   1     1   JCK-2
......................uggCucuuguuugacagcacuc..............................................................................................................................................................................................................   33    1   JCK-3
......................uggaucuuguuuAacagcacuc..............................................................................................................................................................................................................   1     1   JCK-1
......................Cggaucuuguuugacagcacuc..............................................................................................................................................................................................................   5     1   JCK-3
......................uggCucuuguuugacagcacuc..............................................................................................................................................................................................................   36    1   JCK-2
......................uggNucuuguuugacagcacuc..............................................................................................................................................................................................................   7     1   JCK-1
......................uggaucuuguuugCcagcacuc..............................................................................................................................................................................................................   3     1   LCK-3
......................uggaucuuguuugacagcacuG..............................................................................................................................................................................................................   7     1   LT-1
......................uAgaucuuguuugacagcacuc..............................................................................................................................................................................................................   7     1   JCK-2
......................uggaCcuuguuugacagcacuc..............................................................................................................................................................................................................   1     1   LT-2
......................uggaucuuguuugacagUacuc..............................................................................................................................................................................................................   1     1   JCK-1
......................uggCucuuguuugacagcacuc..............................................................................................................................................................................................................   4     1   LT-1
......................uggaucuuguuugacGgcacuc..............................................................................................................................................................................................................   1     1   LT-1
......................uggaucuuguuugacagcacuU..............................................................................................................................................................................................................   22    1   LCK-2
......................uggaucuuAuuugacagcacuc..............................................................................................................................................................................................................   4     1   JCK-2
......................uggCucuuguuugacagcacuc..............................................................................................................................................................................................................   3     1   LCK-3
......................ugAaucuuguuugacagcacuc..............................................................................................................................................................................................................   1     1   LT-1
......................uggaucuuguuugacagcacGc..............................................................................................................................................................................................................   1     1   JCK-3
......................uggaucuuguuuAacagcacuc..............................................................................................................................................................................................................   3     1   JCK-2
......................Cggaucuuguuugacagcacuc..............................................................................................................................................................................................................   6     1   JCK-2
......................uggaucuuguuugacagcacuA..............................................................................................................................................................................................................   59    1   JCK-2
......................uNgaucuuguuugacagcacuc..............................................................................................................................................................................................................   17    1   JCK-2
......................uggaucuuguuugacagcacuG..............................................................................................................................................................................................................   2     1   JCK-3
......................uggaucuuguuugacagcacuU..............................................................................................................................................................................................................   18    1   LCK-1
......................uggaucuuguuugacagcNcuc..............................................................................................................................................................................................................   7     1   JCK-1
......................uggaucuuguuugacagcCcuc..............................................................................................................................................................................................................   9     1   JCK-3
......................uggaucuuguuugacaAcacuc..............................................................................................................................................................................................................   4     1   JCK-1
......................uAgaucuuguuugacagcacuc..............................................................................................................................................................................................................   10    1   JCK-3
......................uggaucuuguuugacagcacuc..............................................................................................................................................................................................................   520   0   LCK-3
......................uggaucuuguuugacaAcacuc..............................................................................................................................................................................................................   3     1   JCK-2
......................uggaucuuguuugacaCcacuc..............................................................................................................................................................................................................   1     1   JCK-3
```

Mature / Star			
cccaccucucuuguuuaccuucuggaucuuguuugacagcacucccaacguuguuguuuggaugacauagaagugcugucgaacaugauccagaaggugcugucgaacaacauacaaaaagggguuuacuuuacguaccgcccaucacgaaaaaaaaaaaaaaaauuguuuaauauuauuucaagauuuauuugaaugaaaauagaacagcuauuccuugguuucuuuaauuacuaaaacccuaugaucuua			
......................uggaucuuguuugacagcacuc...	220	0	JT-1
......................Cggaucuuguuugacagcacuc...	1	1	LT-2
......................uggaucuuguuugacagcacuc...	1262	0	LT-1
......................uAgaucuuguuugacagcacuc...	1	1	LCK-3
......................uggaucuuguuugacagcGcuc...	1	1	JCK-1
......................uggaucCuguuugacagcacuc...	5	1	JCK-3
......................uggaucuuguuugacagcacuU...	48	1	LT-3
......................uggaucuuguuugacagcacuG...	3	1	LT-3
......................uggaucuuguuugacagcacuU...	27	1	LT-2
......................uggCucuuguuugacagcacuc...	7	1	LT-3
......................uUgaucuuguuugacagcacuc...	1	1	JCK-2
......................uggaucuuguuugacagcacuc...	12872	0	JCK-3
......................uggaucuuguuugacGgcacuc...	2	1	JCK-3
......................ugUaucuuguuugacagcacuc...	1	1	JCK-1
......................Cggaucuuguuugacagcacuc...	1	1	LT-1
......................uggaucuCguuugacagcacuc...	2	1	JCK-1
......................uggaucuuguuugacCgcacuc...	5	1	JCK-1
......................uggaucuuguuugacagcCcuc...	7	1	JCK-2
......................uggaucuuguuugacaAcacuc...	6	1	JCK-3
......................ugCaucuuguuugacagcacuc...	1	1	JCK-2
......................uggaucuuUuuugacagcacuc...	1	1	JCK-3
......................ugUaucuuguuugacagcacuc...	1	1	JCK-2
......................uAgaucuuguuugacagcacuc...	1	1	LT-3
......................uggaucuuguuugacagcacuc...	138	0	JT-2
......................uggaCcuuguuugacagcacuc...	9	1	JCK-3
......................uggGucuuguuugacagcacuc...	4	1	JCK-2
......................ugAaucuuguuugacagcacuc...	1	1	LT-2
......................ugAaucuuguuugacagcacuc...	4	1	JCK-2
......................uggaucuuguuugacagcacuc...	76	0	LCK-1
......................uggaucuuguuugacagcaNuc...	11	1	JCK-1
......................uggaucuuguuugacagcacuU...	64	1	LCK-3
......................uggaucuuguuugacagcacuUc...	5	1	LCK-3
......................uggaucuuguuugacagcacucA...	10	1	JT-2
......................uggaucuuguuugacagcacucG...	5	1	JCK-2
......................uggaucuuguuugacagcacuAc...	7	1	LCK-3
......................uggaucuuguuugacagcacucA...	140	1	JCK-2
......................uggaucuuguuugacagcacucA...	14	1	JT-3
......................uggaucuuguuugacagcacuAc...	2	1	LT-2
......................uggaucuuguuugacagcacucA...	6	1	LT-3
......................uggaucuuguuugacagcacuGc...	9	1	LT-1
......................uggaucuuguuugacagcacuUc...	5	1	LT-1
......................uggaucuuguuugacagcacucA...	100	1	JCK-3
......................uggaucuuguuugacagcacucc...	2	0	LT-2
......................uggaucuuguuugacagcacucG...	4	1	JCK-3
......................uggaucuuguuugacagcacucA...	23	1	LT-1
......................uggaucuuguuugacagcacuUc...	6	1	LT-2
......................uggaucuuguuugacagcacuGc...	2	1	LT-2
......................uggaucuuguuugacagcacucG...	3	1	JCK-1
......................uggaucuuguuugacagcacuUc...	13	1	JCK-2
......................uggaucuuguuugacagcacuUc...	11	1	JCK-3
......................uggaucuuguuugacagcacuAc...	6	1	LT-1
......................uggaucuuguuugacagcacucA...	14	1	LT-2
......................uggaucuuguuugacagcacuUc...	1	1	LT-3
......................uggaucuuguuugacagcacuUc...	5	1	JCK-1
......................uggaucuuguuugacagcacuGc...	1	1	LT-3
......................uggaucuuguuugacagcacucA...	79	1	JCK-1
......................uggaucuuguuugacagcacucA...	24	1	LCK-3
......................uggaucuuguuugacagcacuGc...	17	1	LCK-3
......................uggaucuuguuugacagcacuAc...	1	1	JCK-2
......................uggaucuuguuugacagcacuccU...	1	1	LT-1
......................uggaucuuguuugacagcacucAc...	13	1	LCK-3
......................uggaucuuguuugacagcacuccc...	2	0	LT-3
......................uggaucuuguuugacagcacucAc...	2	1	LT-3
......................uggaucuuguuugacagcacucAc...	7	1	LT-1
.......................ggaucuuguuugacagca...	1	0	JCK-1
.......................ggaucuuguuugacagca...	1	0	LT-1
.......................ggaucuuguuugacagca...	2	0	JCK-3
.......................ggaucuuguuugacagcac...	1	0	LT-2
.......................ggaucuuguuugacagcac...	4	0	LCK-3
.......................ggaucuuguuugacagcac...	2	0	LT-1

Mature (uggaucuuguuugacagcacu) **Star** (ugcugucgaacaugauccaga)

Sequence	Reads	Mismatches	Library
cccaccucucuuguuuaccuucuggaucuuguuugacagcacucccaacguuguuguuuggaugacauagaagugcugucgaacaugauccagaaggugcugucgaacaacauacaaaaaggguuuacuuuacguaccgcccaucacgaaaaaaaaaaaaaauuguuuaauauuauuucaagauuuauuugaaugaaaauagaacagcuauuccuugguuucuuuaauuacuaaaacccuaugaucuua			
.......................ggaucuuguuugacagcacu	5	0	LT-2
.......................ggaucuuguuugacagcacu	2	0	LT-3
.......................ggaucuuguuugacagcacu	4	0	JCK-3
.......................ggaucuuguuugacagcacu	5	0	LT-1
.......................ggaucuuguuugacagcacu	3	0	JCK-2
.......................ggaucuuguuugacagcacu	5	0	JCK-1
.......................ggaucuuguuugacagcacu	2	0	LCK-3
.......................ggaucuuguuugacagcacuc	6	0	JCK-2
.......................ggaucuuguuugacagcacuc	6	0	JCK-1
.......................ggaucuuguuugacagcacuc	14	0	JCK-3
.......................ggaucuuguuugacagcacucA	2	1	JCK-2
.......................ggaucuuguuugacagcacuccc	1	0	LT-1
........................gaucuuguuugacagcacu	1	0	LT-1
........................gaucuuguuugacagcacu	14	0	LT-2
........................gaucuuguuugacagcacu	3	0	JCK-1
........................gaucuuguuugacagcacu	3	0	LT-3
........................gaucuuguuugacagcacu	1	0	JCK-2
........................gaucuuguuugacagcacuc	3	0	LT-3
........................gaucuuguuugacagcacuc	2	0	LT-1
........................gaucuuguuugacagcacucc	6	0	LT-2
........................gaucuuguuugacagcacucc	8	0	LT-1
........................gCucuuguuugacagcacucc	1	1	JCK-3
........................gaucuuguuugacagcacucA	1	1	LT-1
........................gaucuuguuugacagcacucc	32	0	JCK-2
........................gaucuuguuugacagcacucc	24	0	JCK-1
........................gaucuuguuugCcagcacucc	1	1	LT-3
........................gaucuuguuugacagcacucc	9	0	LT-3
........................gaucuuguuugacagcacucc	8	0	LCK-3
........................gaucuuguuugacagcacucc	26	0	JCK-3
.........................aucuuguuugacagcacu	3	0	LT-2
.........................aucuuguuugacagcacu	2	0	LT-1
.........................aucuuguuugacagcacuU	1	1	LT-2
.........................aucuuguuugacagcacuc	1	0	JCK-2
.........................aucuuguuugacagcacuc	5	0	LT-3
.........................aucuuguuugacagcacuc	2	0	LT-1
.........................aucuuguuugacagcacuc	8	0	LCK-3
.........................aucuuguuugacagcacuc	1	0	LT-2
.........................aucuuguuugacagcacucc	12	0	JCK-2
.........................aucuuguuugacagcacucc	4	0	LT-1
.........................aucuuguuugacCgcacucc	1	1	JCK-3
.........................aucuuguuugacagcacucc	12	0	LT-3
.........................aucuuguuugacagcacucc	17	0	JCK-3
.........................aucuuguuugacagcacucc	25	0	LCK-2
.........................aucuuguuugacagcacucA	1	1	LT-2
.........................aucuuguuugacagcacucA	7	1	LCK-3
.........................aucuuguuugacagcCcucc	1	1	LT-2
.........................aucuuguuugacagcacucc	9	0	JCK-1
.........................aucuuguuugacagcacucc	7	0	LT-2
.........................aucuuguuugacagcacucc	11	0	LCK-3
.........................aucuuguuugacagcacucA	2	1	LT-1
.........................aucuuguuugacagcacucc	23	0	LCK-1
.........................aucuuguuugacagcacuccc	85	0	JCK-2
.........................aucuuguuugacagcacuccc	39	0	LT-1
.........................aucuuguuugacCgcacuccc	1	1	JCK-3
.........................aucuuguuugacagcacuccc	17	0	JT-1
.........................aucuuguuugacagcacuccc	58	0	LT-2
.........................aucuuguuugacagcacuccc	5	0	LCK-2
.........................aucuuguuugacagcacuccc	58	0	JCK-3
.........................aucuuguuugacagcacuccc	50	0	LT-3
.........................aucuuguuugacCgcacuccc	1	1	JCK-2
.........................aucuuguuugacCgcacuccc	1	1	LT-2
.........................aucuuguuugacagcacuccc	12	0	LCK-1
.........................aucuuguuugacagcacuccc	52	0	JCK-1
.........................aucuuguuugacagcacuccA	1	1	LT-3
.........................aucuuguuugacagcacuccc	32	0	LCK-3
.........................Cucuuguuugacagcacuccc	1	1	LCK-2
.........................aucuuguuugaUagcacuccc	1	1	JCK-2
.........................Cucuuguuugacagcacuccc	1	1	JCK-1
.........................aucuuguuugacagcacucccU	1	1	JCK-2
.........................aucuuguuugacagcacucccU	1	1	JCK-1

Mature **Star**

```
cccaccucucuuguuuaccuucuggaucuuguuugacagcacucccaacguuguuguuuggaugacauagaagugcugucgaacaugauccagaaggugcugucgaacaacauacaaaaaggguuuacuuuacguaccgcccaucacgaaaaaaaaaaaaaauuguuuaauauuauuucaagauuuauuugaaugaaaauagaacagcuauuccuugguuucuuuaauuacuaaaacccuaugaucuua
.........................aucuuguuugacagcacucccU.........   1   1   JCK-3
.........................aucuuguuugacagcacuccca.........   2   0   JCK-2
.........................aucuuguuugacagcacucccU.........   1   1   LT-3
..........................ucuuguuugacagcacuc.........   1   0   LT-2
..........................ucuuguuugacagcacuc.........   2   0   LT-3
..........................ucuuguuugacagcacucc.........   1   0   LT-1
..........................ucuuguuugacagcaUucc.........   1   1   JCK-1
..........................ucuuguuugacagcacucA.........   1   1   LT-3
..........................ucuuguuugacagcaUucc.........   1   1   JCK-2
..........................ucuuguuugacagcacucc.........   3   0   LT-2
..........................ucuuguuugacagcacucc.........   2   0   LT-3
..........................ucuuguuugacagcacuccc.........   2   0   LT-2
..........................ucuuguuugacagcacuccc.........   7   0   JCK-3
..........................ucuuguuugacagcacuccc.........   10  0   JCK-2
..........................ucuuguuugacagcacuccc.........   6   0   JCK-1
..........................ucuuguuugacagcacuccc.........   14  0   LT-1
..........................ucuuguuugacagcacuccU.........   1   1   LT-3
..........................ucuuguuugacagcacuccc.........   5   0   LCK-3
..........................ucuuguuugacagcacuccU.........   1   1   JCK-2
..........................ucuuguuugacagcacuccc.........   18  0   LT-3
..........................ucuuguuugacagcacuccca.........   1   0   JCK-2
..........................ucuuguuugacagcacuccca.........   1   0   JCK-3
..........................ucuuguuugacagcacuccca.........   1   0   LT-1
..........................ucuuguuugacagcacuccca.........   3   0   LT-3
..........................ucuuguuugacagcacuccca.........   1   0   LT-2
..........................ucuuguuugacagcacucccaacg.........   3   0   LCK-3
..........................ucuuguuugacagcacucccaacg.........   1   0   JCK-1
..........................ucuuguuugacagcacucccaacg.........   2   0   LT-2
..........................ucuuguuugacagcacucccaacg.........   1   0   JCK-2
............................uuguuCgacagcacucccaacg.........   1   1   JCK-3
............................uuguuugacagcacucccaacguug.........   2   0   LT-2
...............................uuugacagcacucccaac.........   1   0   LT-1
...............................uuugacagcacucccaac.........   1   0   LT-3
...............................uuugacagcacucccaacg.........   4   0   LCK-3
...............................uuugacagcacucccaacg.........   1   0   JCK-1
...............................uuugacagcacucccaacg.........   2   0   LT-3
...............................uuCgacagcacucccaacgu.........   2   1   JCK-3
...............................uuugacagcacucccaacgu.........   1   0   LT-2
...............................uuCgacagcacucccaacguu.........   1   1   JCK-2
...............................uuugacagcacucccaacguu.........   1   0   LT-3
...............................uuugacagcacucccaacguug.........   1   0   JCK-2
...............................uuugacagcacucccaacguug.........   1   0   JCK-3
...........................................cccaacguuguuguuuggaugacauagaag.........   2   0   JCK-2
...........................................cccaacguuguuguuuggaugacauagaag.........   1   0   LT-2
...........................................cccaacguuguuguuuggaugacauagaag.........   4   0   JCK-3
...........................................cccaacguuguuguuuggaugacauagaag.........   1   0   JCK-1
...................................................uguuguuuggaugacauagaagugc.........   2   0   JCK-1
.....................................................uuguuuggaugacauagaagug.........   1   0   LT-3
.....................................................uuguuuggaugacauagaagugcug.........   2   0   LCK-3
.....................................................uuguuuggaugacauagaagugcug.........   2   0   LT-1
.....................................................uuguuuggaugacauagaagugcugucg.........   1   0   LT-2
.....................................................uuguuuggaugacauagaagugcugucgaa.........   1   0   LT-2
......................................................uguuuggaugacauagaag.........   2   0   LT-1
......................................................uguuuggaugacauagaagugcuguc.........   6   0   JCK-1
........................................................uuuggaugacauagaagugc.........   1   0   LT-1
........................................................uuuggaugacauagaagugcuguc.........   1   0   LT-2
........................................................uuuggaugacauagaagugcugucgaac.........   2   0   LT-1
........................................................uuuggaugacauagaagugcugucgaac.........   1   0   LT-2
........................................................uuuggaugacauagaagugcugucgaacC.........   1   1   LT-1
.........................................................uuggaugacauagaagug.........   2   0   LT-3
.........................................................uuggaugacauagaagugcugucga.........   2   0   LT-1
.........................................................uuggaugacauagaagugcugucgaacU.........   1   1   LT-3
.........................................................uuggaugacauagaagugcugucgaaca.........   1   0   JCK-2
..........................................................uggaugacauagaagugcugucgaa.........   4   0   LCK-3
...........................................................ggaugacauagaagugcugucgaaca.........   1   0   JCK-2
...........................................................ggaugacauagaagugcugucgaaca.........   3   0   JCK-3
............................................................gaugacauagaagugcugu.........   1   0   LT-3
.............................................................augacauagaagugcugucgaacU.........   1   1   JCK-1
..............................................................ugacauagaagugcugucgaa.........   1   0   LT-2
................................................................acauagaagugcugucgaac.........   3   0   JCK-1
```

Mature Star

cccaccucucuuguuuaccuucuggaucuuguuugacagcacucccaacguuguuguuuggaugacauagaagugcugucgaacaugauccagaaggugcugucgaacaacauacaaaaaggggguuuacuuuacguaccgcccaucacgaaaaaaaaaaaaaauuguuuaauauuauuucaagauuuauuugaaugaaaauagaacagcuauuccuugguuucuuuaauuacuaaaacccuaugaucuua			
....acauagaagugcugucgaac	3	0	JCK-2
....acauagaagugcugucgaac	3	0	LT-2
....acauagaagugcugucgaac	2	0	JCK-3
....acauagaagugcugucgaaca	1	0	LT-3
....acauagaagugcugucgaaca	14	0	LCK-3
....acauagaagugcugucgGacaug	6	1	JT-3
....acauagaagugcugucgaacaug	1	0	LT-1
....acauagaagugcugucgGacaug	9	1	JT-2
....acauagaagugcugucgaacauga	1	0	LT-3
....cauagaagugcugucgaaA	2	1	JT-3
....cauagaagugcugucgaaA	4	1	JT-2
....cauagaagugcugucgaacauU	6	1	LCK-3
....auagaagugcugucgaac	1	0	LT-3
....auagaagugcugucgaacU	1	1	JCK-3
....auagaagugcugucgGacaugauc	1	1	JCK-2
....auagaagugcugucgaacaugauc	1	0	LT-2
....auagaagugcugucgaacaugauc	1	0	JCK-1
....auagaagugcugucgaacaugauc	1	0	JCK-3
....auagaagugcugucgaacaugauc	1	0	LT-1
....auagaagugcugucgaacaugauc	2	0	JCK-2
....auagaagugcugucgaacaugaucA	1	1	LT-1
....auagaagugcugucgaacaugauccaga	1	0	JCK-2
....auagaagugcugucgaacaugauccaga	1	0	JCK-3
....uagaagugcugucgGacaugauc	1	1	JCK-2
....gaagugcugucgaacauga	2	0	LT-2
....gaagugcugucgGacaugauc	1	1	JCK-2
....gaagugcugucgGacaugauc	1	1	JCK-1
....gaagugcugucgaacaugaucca	2	0	LT-2
....aagugcugucgaacauga	5	0	JT-2
....aagugcugucgaacauga	17	0	LCK-1
....aagugcugucgaacauga	2	0	LT-3
....aagugcugucgaacauga	17	0	LCK-2
....aagugcugucgaacauAa	2	1	LCK-3
....aagugcugucgaacauga	5	0	LCK-3
....aagugcugucgaacauga	2	0	JCK-3
....aagugcugucgaacauga	6	0	JT-3
....aagugcugucgaacauga	12	0	JT-1
....aagugcugucgaacauga	1	0	JCK-2
....aagugcugucgaacaugUu	1	1	JCK-3
....aagugcugucgaacaugUu	6	1	LT-2
....aagugcugucgaacaugau	4	0	LT-1
....aagugcugucgaacaugau	1	0	LT-2
....aagugcugucgaacaugaA	1	1	LT-3
....aagugcugucgaacaugau	6	0	LCK-3
....aagugcugucgaacaugau	1	0	LT-3
....aagugcugucgGacaugau	2	1	JCK-2
....aagugcugucgaacaugUu	3	1	LCK-3
....aagugcugucgaacaugUu	1	1	JCK-1
....aagugcugucgGacaugau	1	1	JCK-3
....aagugcugucgGacaugau	1	1	JCK-1
....aagugcugucgaCcaugauc	1	1	LT-3
....aagugcugucgaacaugauc	11	0	LCK-3
....aagugcugucgaacaugauc	24	0	LT-3
....aagugcugucgaacaugauc	24	0	LT-1
....aagugcugucgGacaugauc	119	1	JCK-2
....aagugcugucgaacaugauc	26	0	JCK-2
....aagugcugucgGacaugauc	73	1	JCK-1
....aagugcugucgaacaugauc	19	0	LT-2
....aagugcugucgaacaugauc	19	0	JT-3
....aagugcugucgaacaugauc	17	0	JCK-3
....aagugcugucgaacaugauc	7	0	JT-2
....aagugcugucgaacauAauc	1	1	JCK-2
....aagugcugucgaacaugauU	1	1	LT-1
....aagugcugucgaacaugauN	1	1	JCK-1
....aagugcugucgaacaugauc	15	0	JCK-1
....aagugcugucgaacauAauc	1	1	JCK-3
....aagugcugucgGacaugauc	84	1	JCK-3
....aagugcugucgaacaugauU	5	1	LT-2
....aagugcugucgGacaugauc	37	1	JT-1
....aagugcugucgaacaugaucc	64	0	JT-1

Mature **Star**

cccaccucucuuguuuaccuucuggaucuuguuugacagcacucccaacguuguuguuuggaugacauagaagugcugucgaacaugauccagaaggugcugucgaacaacauacaaaaaggggguuuacuuuacguaccgcccaucacgaaaaaaaaaaaaaaauuguuuaauauuauuucaagauuuauuugaaugaaaauagaacagcuauuccuugguuucuuuaauuacuaaaacccuaugaucuua

aagugcugucgUacaugaucc	1	1	JCK-3
aagugcugucgaacaugaucc	5	0	JT-2
Cagugcugucgaacaugaucc	1	1	LCK-3
Cagugcugucgaacaugaucc	1	1	JCK-2
aagugcugucgaacaugaucc	74	0	JCK-2
aagugcugucgGacaugaucc	13	1	JT-1
aagugcugucgaacaugaucc	57	0	LT-1
aagugcugucgaacaugaucc	63	0	LCK-3
aagugcugucgaacaugaucc	22	0	LCK-1
aagugcugucgaacaugaucA	1	1	LT-3
aagugcugucgaacaugaucc	1	0	JT-3
aagugcugucgaacaugCucc	1	1	JCK-1
aagugcugucgGacaugaucc	248	1	JCK-2
aagugcugucgGacaugaucc	17	1	JT-3
aagugcugucgGacaugaucc	191	1	JCK-3
aagugcugucgGacaugaucc	8	1	JT-2
Cagugcugucgaacaugaucc	1	1	LT-1
aagugcugucgaacaugaucc	64	0	JCK-3
aagugcugucgGacaugaucc	137	1	JCK-1
aagGgcugucgaacaugaucc	1	1	JCK-1
aagugcugucgaacaugaucc	106	0	LT-3
aagugcugucgaacaugaucc	26	0	LCK-2
aagugcugucgaacaugaucU	1	1	JCK-2
aagugcugucgaaNaugaucc	1	1	LCK-1
aagugcugucgaacNugaucc	1	1	LCK-1
aagugcugucgaacaugaucc	57	0	JCK-1
aagugcugucgaacaugaucc	94	0	LT-2
aCgugcugucgaacaugaucc	4	1	LT-2
aagugcugucgaacaugaucU	2	1	JCK-3
Cagugcugucgaacaugaucca	1	1	JCK-3
aagugcugucgaacaugaucca	4	0	LT-2
aagugcugucgaacaugaucca	1	0	LT-3
aagugcugucgaacaugauccag	5	0	LT-1
aagugcugucgaacaugauccag	8	0	JCK-2
aagugcugucgaacaugauccag	7	0	JCK-3
aagugcugucgGacaugauccag	2	1	JCK-3
aagugcugucgaacaugauccaA	3	1	LT-1
aagugcugucgaacaugauccag	4	0	JCK-1
aagugcugucgGacaugauccag	3	1	JCK-1
aagugcugucgaacaugauccag	2	0	LT-3
aagugcugucgaacaugauccaA	3	1	JCK-1
aagugcugucgGacaugauccag	4	1	JCK-2
aagugcugucgaacaugauccag	2	0	LT-2
aagugcugucgaCcaugauccaga	1	1	LT-2
aagugcugucgaacaugauccagU	2	1	JCK-2
aagugcugucgGacaugauccaga	48	1	JCK-2
aagugcugucgaacaugauccaga	42	0	JCK-3
aagugcugucgaacaugauccaga	21	0	LT-3
aNgugcugucgaacaugauccaga	1	1	JCK-3
aagugcugucgaacaugauccaga	7	0	JT-1
aCgugcugucgaacaugauccaga	1	1	JCK-3
aagugcugucgaacaugauAcaga	1	1	JCK-1
aagugcugucgGacaugauccaga	27	1	JCK-1
aagugcugucgaacaugauccaga	15	0	LT-2
aagugcugucgaacaugauccaga	13	0	LCK-3
aagugcugucgaacauUauccaga	1	1	JCK-2
aagugcugucgaacaugauccagU	2	1	JCK-1
aagugcugucgaacaugauccaga	71	0	JCK-2
aagugcugucgGacaugauccaga	39	1	JCK-3
aagugcugucgaacaugauccagU	1	1	JCK-3
aagugcugucgaaAaugauccaga	1	1	LT-2
aagugcugucgaacaugauccaga	26	0	LT-1
aagugcugucgaacaugauccaga	34	0	JCK-1
agugcugucgaacaugUu	9	1	LT-3
agugcugucgaacauAau	7	1	LCK-3
agugcugucgaacaugau	1	0	JCK-2
agugcugucgaacGugau	1	1	LCK-1
agugcugucgaacaugUu	22	1	LCK-3
agugcugucgaacaugaC	1	1	JCK-3
agugcugucgaacaugau	16	0	LCK-2

Mature **Star**

cccaccucucuuguuuaccuucuggaucuuguuugacagcacucccaacguuguuguuuggaugacauagaagugcugucgaacaugauccagaaggugcugucgaacaacauacaaaaaggggguuuacuuuacguaccgcccaucacgaaaaaaaaaaaaaaauuguuuaauauuauuucaagauuuauuugaaugaaaauagaacagcuauuccuugguuucuuuaauuacuaaaacccuaugaucuua

agugcugucgaacaugau	17	0	LCK-1
agugcugucgaacaugau	19	0	LT-2
agugcugucgaacaugUu	1	1	LT-1
agugcugucgaacaugau	5	0	LT-3
agugcugucgaacaugUu	8	1	LT-2
agugcugucgaacaugUu	1	1	JCK-3
agugcugucgCacaugau	1	1	LCK-2
agugcugucgaacaugau	4	0	LT-1
agugcugucgaacCugauc	1	1	LCK-1
agugcugucgaacaugauU	13	1	LT-2
agugcugucgaacaugauc	63	0	JCK-1
agugcugucgGacaugauc	10	1	JCK-2
agugcugucgaacaugauc	68	0	JCK-3
agugcugucgaacaugauc	15	0	LCK-1
agugcugucgaacaugauU	16	1	LT-3
agugcugucgaacaugauc	17	0	LCK-3
agugcugucgGacaugauc	5	1	JCK-1
agugcugucgaacaugauU	7	1	JCK-2
agNgcugucgaacaugauc	1	1	LT-2
agugcugucgaacaugauU	3	1	JCK-1
agugcugucgaacaugauc	69	0	LT-2
agugcugucgaacaugauc	88	0	LT-3
agugcugucgaacaugauA	1	1	LT-1
agugcugucgaacaugauc	24	0	LCK-2
agugcugucgaNcaugauc	1	1	JCK-1
agugcugucgaacaugauU	4	1	LT-1
agugcugucgaacaugauU	15	1	LCK-3
agugcugucgaacaugauc	42	0	LT-1
agugcugucgaacaugauc	1	0	JT-3
agugcugucgaacaugauc	94	0	JCK-2
agugcugucgGacaugauc	3	1	JCK-3
agugcugucgaacaugaucA	4	1	JT-3
aAugcugucgaacaugaucc	1	1	LT-2
agugcugucgaCcaugaucc	1	1	LT-3
agugcugucgaacaugaucA	1	1	LT-3
agugcugucgaacaugaucc	38	0	JCK-3
agugcugucgaacCugaucc	1	1	LT-2
agugcugucgaacaugauUc	1	1	LT-1
agugcugucgaacaugaucc	22	0	LCK-1
agugcugucgaacaugaucc	26	0	JCK-1
agugcugucgaacaugaucc	72	0	LT-1
agugcugucgaacaugaucc	90	0	LCK-3
agugcugucgaacaugaucc	130	0	LT-3
agugcugucgGacaugaucc	9	1	JCK-2
agugcugucgaacaugCucc	2	1	LT-2
agugcugucgaacaugaucA	4	1	JT-2
agugcugucgaacaugaucc	5	0	JT-2
agugcugucgGacaugaucc	3	1	JCK-3
agugcugucgaacaugaucc	118	0	LT-2
agugcugucgaacaugaucc	8	0	JT-3
agugcugucgGacaugaucc	7	1	JCK-1
agugcugucgaacaugaucU	4	1	LT-3
Cgugcugucgaacaugaucc	1	1	LT-1
agugcuguAgaacaugaucc	1	1	JCK-2
agugcugucgaacaugaucU	2	1	LT-1
agugcugucgaacaugaucA	1	1	JCK-3
aguAcugucgaacaugaucc	1	1	LT-2
agugcugucgaacaugauUc	1	1	LT-3
agugcugucgaacaugaucU	2	1	LT-2
agugcugucgaacaugaucc	49	0	JCK-2
agugcugucgaacaugaucc	21	0	LCK-2
agugcugucgaacaugauccU	2	1	JCK-1
agugcugucgaacaugauccC	2	1	JCK-3
agugcugucgaacaugaucca	115	0	LT-1
agugcugucgaacaugaucNa	1	1	JCK-1
agugcugucgaacaugaucca	152	0	JCK-1
agugcugucgaacaugauccC	6	1	JCK-2
agugcugucgaacaugaucca	226	0	JCK-2
agugcugucgaacaugauccU	1	1	LT-1
agugcugucgaacaugauccC	1	1	LT-1

Mature **Star**

```
cccaccucucuuguuuaccuucuggaucuuguuugacagcacucccaacguuguuguuuggaugacauagaagugcugucgaacaugauccagaaggugcugucgaacaacauacaaaagggguuuacuuuacguaccgcccaucacgaaaaaaaaaaaaaaaauuguuuaauauuauuucaagauuuauuugaaugaaaauagaacagcuauuccuugguuucuuuaauuacuaaaacccuaugaucuua
........................................................................agugcugucgaacaugauccU....	1	1	JCK-2
........................................................................agugcugucgGacaugaucca....	10	1	JCK-3
........................................................................agugcugucgaacaugaucca....	36	0	LCK-3
........................................................................agugcugucgaacaugauccC....	1	1	JCK-1
........................................................................agugcugucgaacaugauccU....	2	1	LT-2
........................................................................agugcugucgaacaugaucca....	24	0	JT-1
........................................................................aNugcugucgaacaugaucca....	1	1	JCK-3
........................................................................agugcugucgaacaugaucca....	121	0	LT-3
........................................................................agugcugucgaCcaugaucca....	1	1	JCK-1
........................................................................agugcugucgaacaugaucca....	43	0	LCK-1
........................................................................agugcugucgaCcaugaucca....	1	1	LT-2
........................................................................agugcugucgaacaugCucca....	1	1	LCK-3
........................................................................agugcugucgaacaugaucca....	144	0	JCK-3
........................................................................agugcugucgaacaugaucca....	53	0	LCK-2
........................................................................agugcugucgGacaugaucca....	19	1	JCK-2
........................................................................agugcugucgaacaGgaucca....	1	1	LCK-1
........................................................................agugcugucgaacaugauccC....	3	1	LT-3
........................................................................agugcugucgaacaugauccU....	3	1	JCK-3
........................................................................agugcugucgGacaugaucca....	13	1	JCK-1
........................................................................agugcugucgaacaugaucca....	93	0	LT-2
........................................................................agugcugucgaacaugauccag....	2	0	JCK-1
........................................................................agugcugucgaacaugauccaga....	3	0	JCK-1
........................................................................agugcugucgGacaugauccaga....	3	1	JCK-2
........................................................................agugcugucgGacaugauccaga....	2	1	JCK-3
........................................................................agugcugucgGacaugauccaga....	1	1	JCK-1
........................................................................agugcugucgaacaugauccaga....	2	0	JCK-2
........................................................................agugcugucgaacaugauccaga....	2	0	JCK-3
.........................................................................gugcugucgGacaugauc....	3	1	JCK-1
.........................................................................gugcugucgGacaugauc....	5	1	JCK-3
.........................................................................gugcugucgaacaugauA....	1	1	LT-1
.........................................................................gugcugucgGacaugauc....	6	1	JCK-2
.........................................................................gugcugucgaacaugauA....	1	1	LT-3
.........................................................................gugcugucgaCcaugaucc....	1	1	LT-2
.........................................................................gugcugucgaacaugauUc....	1	1	LT-1
.........................................................................gugcugucgGacaugaucca....	1	1	JCK-2
.........................................................................gugcugucgaacaugaucca....	7	0	JCK-1
.........................................................................gugcugucgaGcaugaucca....	1	1	LT-3
.........................................................................gugcugucgaacaugaucca....	12	0	LT-1
.........................................................................gugcugucgaacaugaucca....	4	0	JCK-3
.........................................................................gugcugucgaacaugaucca....	16	0	LT-3
.........................................................................gugcugucgaacaugaucca....	18	0	LT-2
.........................................................................gugcugucgaacaugaucca....	9	0	LCK-3
.........................................................................gugcugucgaacaugaucca....	14	0	JCK-2
.........................................................................gugcugucgaacaugauccU....	1	1	LT-2
.........................................................................gugcugucgaacaugauccag....	14	0	LT-3
.........................................................................gugcugucgGacaugauccag....	5	1	JCK-2
.........................................................................gugcugucgaacaugauccaA....	1	1	JCK-2
.........................................................................gugcugucgaacaugauccag....	15	0	JCK-2
.........................................................................gugcugucgGacaugauccag....	2	1	JCK-3
.........................................................................Cugcugucgaacaugauccag....	1	1	LT-3
.........................................................................gugcugucgaacaugauccag....	10	0	LT-2
.........................................................................gugcugucgGacaugauccag....	2	1	JCK-1
.........................................................................gugcugucgaacaugauccag....	17	0	LT-1
.........................................................................gugcugucgaacaugauccag....	24	0	JCK-3
.........................................................................gugcugucgaacaugauccag....	9	0	JCK-1
.........................................................................gugcugucgaacaugauccag....	6	0	LCK-3
.........................................................................gugcugucgaacaugauccagU....	1	1	LT-1
.........................................................................Cugcugucgaacaugauccaga....	2	1	JCK-2
.........................................................................gugcugucgaacaugauccaga....	2	0	JCK-3
.........................................................................gugcugucgaacaugauccaga....	1	0	JCK-2
.........................................................................gugcugucgaacaugauccagU....	1	1	LT-3
.........................................................................gugcugucgaacaugauccaga....	1	0	JCK-1
.........................................................................Cugcugucgaacaugauccaga....	2	1	LT-3
.........................................................................gugcugucgaacaugauccagU....	2	1	JCK-1
.........................................................................gugcugucgGacaugauccaga....	1	1	JCK-2
.........................................................................Cugcugucgaacaugauccaga....	1	1	JCK-3
.........................................................................gugcugucgaacaugauccagU....	1	1	LCK-3
.........................................................................Cugcugucgaacaugauccaga....	2	1	JCK-1
.........................................................................gugcugucgGacaugauccaga....	3	1	JCK-1
..........................................................................ugcugucgaacaugaucU....	2	1	LT-3
```

Mature · Star

```
cccaccucucuuguuuaccuucuggaucuuguuugacagcacucccaacguuguuguuuggaugacauagaagugcugucgaacaugauccagaaggugcugucgaacaacauacaaaaaggguuuacuuuacguaccgcccaucacgaaaaaaaaaaaaaaauuguuuaauauuauuucaagauuuauuugaaugaaaauagaacagcuauuccuugguuucuuuaauuacuaaaacccuaugaucuua
.........................................................................ugcugucgGacaugaucc............................................................................................................................................................... 5    1  JT-2
.........................................................................ugcugucgaacaugaucU............................................................................................................................................................... 19   1  LCK-2
.........................................................................ugcugucgGacaugaucc............................................................................................................................................................... 140  1  JCK-3
.........................................................................ugcugucgGacaugaucc............................................................................................................................................................... 112  1  JCK-1
.........................................................................ugcugucgaacaugaucU............................................................................................................................................................... 2    1  LT-2
.........................................................................ugcugucgaacaugaucU............................................................................................................................................................... 1    1  LT-1
.........................................................................ugcugucgaacaugaucA............................................................................................................................................................... 1    1  LT-3
.........................................................................ugcugucgaacaugaucU............................................................................................................................................................... 31   1  LCK-1
.........................................................................ugcugucgaacaugaucU............................................................................................................................................................... 1    1  JCK-2
.........................................................................ugcugucgaacaugaucA............................................................................................................................................................... 4    1  LT-1
.........................................................................ugcugucgGacaugaucc............................................................................................................................................................... 28   1  JT-1
.........................................................................ugcugucgaacaugaucU............................................................................................................................................................... 16   1  LCK-3
.........................................................................ugcugucgGacaugaucc............................................................................................................................................................... 3    1  JT-3
.........................................................................ugcugucgaacCugaucc............................................................................................................................................................... 1    1  LCK-3
.........................................................................ugcugucgGacaugaucc............................................................................................................................................................... 195  1  JCK-2
.........................................................................ugcugucgaacaugaCcca.............................................................................................................................................................. 1    1  LCK-3
.........................................................................ugcugucgGacaugaucca.............................................................................................................................................................. 7    1  JT-1
.........................................................................ugcugucgaacaugaucca.............................................................................................................................................................. 6    0  JT-2
.........................................................................ugcugucgGacaugaucca.............................................................................................................................................................. 30   1  JCK-3
.........................................................................ugcugucgaacaugaucca.............................................................................................................................................................. 53   0  LCK-1
.........................................................................ugcugucgaacaugaucca.............................................................................................................................................................. 4    0  JT-3
.........................................................................ugcugucgaacaugaucca.............................................................................................................................................................. 96   0  LT-3
.........................................................................ugcugucgGacaugaucca.............................................................................................................................................................. 3    1  JT-3
.........................................................................ugcugucgaacaugauccU.............................................................................................................................................................. 1    1  LT-1
.........................................................................ugcugucgaacGugaucca.............................................................................................................................................................. 1    1  LCK-3
.........................................................................ugcugucgaacaugaucca.............................................................................................................................................................. 81   0  LCK-3
.........................................................................ugcugucgaacaugaucca.............................................................................................................................................................. 61   0  LT-1
.........................................................................ugcugucgaacaugaucca.............................................................................................................................................................. 18   0  JCK-3
.........................................................................ugcugucgaacaugCucca.............................................................................................................................................................. 1    1  LT-2
.........................................................................ugcugucgGacaugaucca.............................................................................................................................................................. 59   1  JCK-2
.........................................................................ugcugucgaacaugaucca.............................................................................................................................................................. 24   0  JCK-1
.........................................................................ugcugucgaacaugaucca.............................................................................................................................................................. 125  0  LT-2
.........................................................................ugcugucgaacaugaucca.............................................................................................................................................................. 67   0  JT-1
.........................................................................ugcugucgGacaugaucca.............................................................................................................................................................. 41   1  JCK-1
.........................................................................uAcugucgaacaugaucca.............................................................................................................................................................. 1    1  LCK-3
.........................................................................ugcugucgaacaugaucca.............................................................................................................................................................. 26   0  JCK-2
.........................................................................ugcugucgaaAaugaucca.............................................................................................................................................................. 1    1  LT-2
.........................................................................ugcugucgaacaugaucca.............................................................................................................................................................. 50   0  LCK-2
.........................................................................ugcugucgaacaugauccag............................................................................................................................................................. 458  0  LT-2
.........................................................................ugcugucgaacaugGuccag............................................................................................................................................................. 1    1  JT-1
.........................................................................ugcugucgaacaugauccaC............................................................................................................................................................. 1    1  LT-3
.........................................................................ugcNgucgaacaugauccag............................................................................................................................................................. 1    1  JCK-1
.........................................................................ugcugucgaacaugauccag............................................................................................................................................................. 487  0  LCK-3
.........................................................................ugcugucgCacaugauccag............................................................................................................................................................. 2    1  LT-2
.........................................................................uAcugucgaacaugauccag............................................................................................................................................................. 1    1  LT-2
.........................................................................uNcugucgaacaugauccag............................................................................................................................................................. 1    1  JCK-3
.........................................................................ugcugucgaacCugauccag............................................................................................................................................................. 2    1  LT-1
.........................................................................ugcugucgaacaugauccaA............................................................................................................................................................. 2    1  LT-3
.........................................................................ugcugucgaacaugauccag............................................................................................................................................................. 143  0  JCK-1
.........................................................................ugcugucgaacaugauccaA............................................................................................................................................................. 2    1  JCK-2
.........................................................................ugcugucgCacaugauccag............................................................................................................................................................. 1    1  LT-1
.........................................................................ugcugucgaacaugauccag............................................................................................................................................................. 45   0  JT-2
.........................................................................ugcugucgaacaugauccaN............................................................................................................................................................. 1    1  JCK-1
.........................................................................ugcugucgaacaugCuccag............................................................................................................................................................. 1    1  LT-1
.........................................................................ugcugucgGacaugauccag............................................................................................................................................................. 497  1  JCK-3
.........................................................................ugcugucgGacaugauccag............................................................................................................................................................. 431  1  JCK-1
.........................................................................ugcugucgCacaugauccag............................................................................................................................................................. 1    1  LCK-3
.........................................................................ugcugucAaacaugauccag............................................................................................................................................................. 1    1  LT-2
.........................................................................ugcugucgaacaugauccCg............................................................................................................................................................. 1    1  LT-3
.........................................................................ugcugucgaacaugauccaA............................................................................................................................................................. 2    1  JCK-3
.........................................................................ugNugucgaacaugauccag............................................................................................................................................................. 1    1  LT-2
.........................................................................ugcugucgaacaugauccag............................................................................................................................................................. 73   0  JT-3
.........................................................................ugcugucgCacaugauccag............................................................................................................................................................. 1    1  JCK-3
.........................................................................ugcugucgGacaugauccag............................................................................................................................................................. 29   1  JT-2
.........................................................................ugcugucgaacCugauccag............................................................................................................................................................. 1    1  LT-2
.........................................................................ugcugucgaacaugCuccag............................................................................................................................................................. 1    1  LT-3
.........................................................................ugcugucgaacaugauccaU............................................................................................................................................................. 1    1  JCK-1
.........................................................................uNcugucgaacaugauccag............................................................................................................................................................. 1    1  JT-1
.........................................................................ugcugucgaCcaugauccag............................................................................................................................................................. 1    1  LT-3
.........................................................................ugcugucgaacaugauccag............................................................................................................................................................. 117  0  LCK-2
```

Mature (red) … **Star** (purple)

cccaccucucuuguuuaccuucuggaucuuguuugacagcacucccaacguuguuguuuggaugacauagaagugcugucgaacaugauccagaaggugcugucgaacaacauacaaaagggguuuacuuuacguaccgcccaucacgaaaaaaaaaaaaaauuguuuaauauuauuucaagauuuauuugaaugaaaauagaacagcuauuccuugguuucuuuaauuacuaaaacccuaugaucuua

ugcugucgaacaugauccag	40	0	JT-1
ugcugucgaCcaugauccag	1	1	LT-2
ugcugucgGacaugauccag	64	1	JT-3
ugcugucgaacaugauccag	441	0	LT-1
ugcugucgaacaugauccag	530	0	LT-3
ugcugCcgaacaugauccag	1	1	LT-3
ugcugucgGacaugauccag	49	1	JT-1
ugcugucgGacaugauccag	787	1	JCK-2
ugcugucgaacCugauccag	1	1	LCK-2
ugcugucgaCcaugauccag	1	1	LCK-3
ugcugucgaacaugauccag	114	0	LCK-1
ugcugucgaacaugauccag	169	0	JCK-3
ugcugucgaacaugauccag	262	0	JCK-2
ugcugucgaacaugCuccag	1	1	LT-2
ugcugAcgaacaugauccag	1	1	LT-3
ugcugucgaCcaugauccag	1	1	LCK-2
ugcugucgaacaugauccaga	107	0	LCK-2
ugcugucgNacaugauccaga	2	1	JCK-3
uAcugucgaacaugauccaga	1	1	LT-2
uAcugucgaacaugauccaga	1	1	LCK-3
ugcugucAaacaugauccaga	1	1	LT-1
ugcuguUgaacaugauccaga	1	1	JCK-2
uUcugucgaacaugauccaga	1	1	JCK-1
ugcugucgaacaugauccagC	1	1	JT-3
ugGugucgaacaugauccaga	1	1	LT-2
ugcNgucgaacaugauccaga	4	1	JCK-1
ugcugucgaacaugauccaga	1673	0	JCK-1
ugAugucgaacaugauccaga	1	1	LCK-3
ugcugucgaGcaugauccaga	1	1	JCK-3
ugcugucgaacaugauccGga	1	1	JCK-3
ugcugucgaacCugauccaga	2	1	LT-3
ugcugucgaacaugauccagU	2	1	JCK-2
ugcugucgaacaugauccagC	1	1	LT-3
ugcugucgaacauAauccaga	2	1	JCK-3
ugcugucgaacaugauccCga	4	1	JCK-2
ugcugCcgaacaugauccaga	2	1	LT-3
ugcugucgaCcaugauccaga	3	1	JCK-1
ugcugucgaacCugauccaga	2	1	LT-2
ugcugucgGacaugauccaga	17	1	JT-2
uUcugucgaacaugauccaga	2	1	LT-2
ugcugucgaacGugauccaga	1	1	JCK-3
ugcugucgaacaugauccaga	186	0	JT-1
ugcugucgCacaugauccaga	10	1	LT-2
uAcugucgaacaugauccaga	4	1	LT-3
ugcugucgaacauAauccaga	2	1	LT-1
ugcuguAgaacaugauccaga	1	1	JCK-2
Cgcugucgaacaugauccaga	1	1	LT-1
ugcugCcgaacaugauccaga	1	1	JCK-3
ugcugucgaacaugauccCga	5	1	LT-2
uAcugucgaacaugauccaga	3	1	LT-1
ugcuguUgaacaugauccaga	1	1	JCK-3
ugcugucgaacGugauccaga	1	1	LCK-3
uUcugucgaacaugauccaga	1	1	JCK-2
ugcCgucgaacaugauccaga	2	1	LT-1
ugcugucgaacauAauccaga	1	1	JCK-2
ugcugucgaCcaugauccaga	1	1	JCK-3
ugcuguAgaacaugauccaga	1	1	LT-2
ugcugucgGacaugauccaga	9455	1	JCK-3
uNcugucgaacaugauccaga	4	1	JCK-2
uAcugucgaacaugauccaga	1	1	JCK-1
ugcugucUaacaugauccaga	1	1	LT-1
ugcugucgaacCugauccaga	1	1	LT-1
ugcugucgaacaugCuccaga	1	1	JCK-1
ugcugucgaacaugauccCga	5	1	LT-3
ugcugucgaacaugauccagC	1	1	JCK-1
ugcugucgaacaugaucAaga	3	1	LT-3
uCcugucgaacaugauccaga	1	1	LT-2
uNcugucgaacaugauccaga	3	1	JCK-1
ugcugucgaacaugauccaAa	5	1	JCK-3
ugcugucgaacaugaucNaga	1	1	JCK-3

```
                      Mature                                                 Star
cccaccucucuuguuuaccuucuggaucuuguuugacagcacucccaacguuguuguuuggaugacauagaagugcugucgaacaugauccagaaggugcugucgaacaacauacaaaaggguuuacuuuacguaccgcccaucacgaaaaaaaaaaaaaaauuguuuaauauuauuucaagauuuauuugaaugaaaauagaacagcuauuccuugguuucuuuaauuacuaaaacccuaugaucuua
.........................................................................ugcugucgCacaugauccaga........................................................................................................................................................... 3      1    JCK-3
.........................................................................ugcugucAaacaugauccaga........................................................................................................................................................... 1      1    JCK-2
.........................................................................ugcugucgaacaugauccaga........................................................................................................................................................... 2141   0    LT-1
.........................................................................ugcugucgaacaugauccaga........................................................................................................................................................... 2163   0    JCK-3
.........................................................................ugcugucgaCcaugauccaga........................................................................................................................................................... 5      1    LT-2
.........................................................................ugcugucgaacaugGuccaga........................................................................................................................................................... 1      1    JCK-3
.........................................................................ugcugucgUacaugauccaga........................................................................................................................................................... 1      1    JCK-2
.........................................................................ugcugucgaacaugCuccaga........................................................................................................................................................... 3      1    LT-3
.........................................................................ugcugucgaacaCgauccaga........................................................................................................................................................... 1      1    LT-1
.........................................................................ugcugucgaacaugauccagU........................................................................................................................................................... 5      1    JCK-1
.........................................................................ugcuguUgaacaugauccaga........................................................................................................................................................... 1      1    LT-1
.........................................................................ugcugucgaCcaugauccaga........................................................................................................................................................... 5      1    LT-3
.........................................................................ugcugucgGacaugauccaga........................................................................................................................................................... 20     1    JT-3
.........................................................................ugcugucgaacGugauccaga........................................................................................................................................................... 1      1    JCK-2
.........................................................................ugcugucgaacauAauccaga........................................................................................................................................................... 3      1    LT-2
.........................................................................ugUugucgaacaugauccaga........................................................................................................................................................... 1      1    LT-3
.........................................................................ugcugucgaacaugaCccaga........................................................................................................................................................... 1      1    LT-3
.........................................................................ugcugucgaacaugaucUaga........................................................................................................................................................... 1      1    LT-3
.........................................................................ugcugucgCacaugauccaga........................................................................................................................................................... 3      1    LT-3
.........................................................................ugcugucgaacNugauccaga........................................................................................................................................................... 4      1    JCK-1
.........................................................................uAcugucgaacaugauccaga........................................................................................................................................................... 1      1    JCK-2
.........................................................................ugcugucAaacaugauccaga........................................................................................................................................................... 2      1    LT-2
.........................................................................ugcugucgaaAaugauccaga........................................................................................................................................................... 1      1    LT-1
.........................................................................ugcugucgGacaugauccaga........................................................................................................................................................... 12782  1    JCK-2
.........................................................................ugcuguAgaacaugauccaga........................................................................................................................................................... 1      1    LT-1
.........................................................................uNcugucgaacaugauccaga........................................................................................................................................................... 3      1    JCK-3
.........................................................................ugcugucgaacCugauccaga........................................................................................................................................................... 5      1    JCK-2
.........................................................................ugcuAucgaacaugauccaga........................................................................................................................................................... 1      1    LT-3
.........................................................................ugcugucgaacaugauccCga........................................................................................................................................................... 1      1    JCK-3
.........................................................................ugcugucgCacaugauccaga........................................................................................................................................................... 1      1    LT-1
.........................................................................ugcugucgaacaugauccagC........................................................................................................................................................... 1      1    JT-1
.........................................................................ugcugucgCacaugauccaga........................................................................................................................................................... 2      1    LCK-3
.........................................................................ugcugucgCacaugauccaga........................................................................................................................................................... 5      1    JCK-2
.........................................................................ugNugucgaacaugauccaga........................................................................................................................................................... 7      1    LT-2
.........................................................................ugcugucgGacaugauccaga........................................................................................................................................................... 7517   1    JCK-1
.........................................................................ugcugucgaacCugauccaga........................................................................................................................................................... 1      1    JCK-1
.........................................................................ugcugucgaacaugauccaNa........................................................................................................................................................... 4      1    JCK-1
.........................................................................ugcugucgaacaugauccaga........................................................................................................................................................... 125    0    LCK-1
.........................................................................ugcugCcgaacaugauccaga........................................................................................................................................................... 1      1    JCK-2
.........................................................................ugcugucgaacaugaucAaga........................................................................................................................................................... 1      1    JCK-2
.........................................................................ugcugucgaacaugauGcaga........................................................................................................................................................... 2      1    JCK-1
.........................................................................uAcugucgaacaugauccaga........................................................................................................................................................... 1      1    JCK-3
.........................................................................ugcugucgaCcaugauccaga........................................................................................................................................................... 1      1    LCK-3
.........................................................................ugcugucgaCcaugauccaga........................................................................................................................................................... 3      1    LT-1
.........................................................................ugcugucgaacaugauccaAa........................................................................................................................................................... 1      1    JCK-2
.........................................................................ugcugucgaacaugauccaga........................................................................................................................................................... 1025   0    LCK-3
.........................................................................ugcugucgaGcaugauccaga........................................................................................................................................................... 1      1    JCK-2
.........................................................................ugcugucgCacaugauccaga........................................................................................................................................................... 3      1    JCK-1
.........................................................................ugcugucgaacaugCuccaga........................................................................................................................................................... 7      1    LT-2
.........................................................................ugcugucgaacaugCuccaga........................................................................................................................................................... 4      1    JCK-3
.........................................................................ugcugucgaacaugCuccaga........................................................................................................................................................... 3      1    JCK-2
.........................................................................ugcugucgaacaugauccaga........................................................................................................................................................... 118    0    JT-2
.........................................................................ugcugucgaacaugauccagC........................................................................................................................................................... 1      1    JCK-2
.........................................................................ugcugucgaacaugauccaga........................................................................................................................................................... 2567   0    LT-3
.........................................................................ugcugucgaCcaugauccaga........................................................................................................................................................... 3      1    JCK-2
.........................................................................ugcugucgaacaugauccaga........................................................................................................................................................... 2228   0    LT-2
.........................................................................ugcugucgaacaugauccaga........................................................................................................................................................... 2920   0    JCK-2
.........................................................................ugcugucgaacaugauccaga........................................................................................................................................................... 145    0    JT-3
.........................................................................ugcugucgGacaugauccaga........................................................................................................................................................... 44     1    JT-1
.........................................................................ugcugucgaacaugauccagaC.......................................................................................................................................................... 3      1    JCK-2
.........................................................................ugcugucgGacaugauccagaa.......................................................................................................................................................... 5      1    JCK-1
.........................................................................ugcugucgaacaugauccagaa.......................................................................................................................................................... 2      0    JCK-2
.........................................................................ugcugucgaacaugauccagaU.......................................................................................................................................................... 4      1    LT-3
.........................................................................ugcugucgaacaugauccagaa.......................................................................................................................................................... 3      0    LT-2
.........................................................................ugcugucgGacaugauccagaa.......................................................................................................................................................... 12     1    JCK-2
.........................................................................ugcugucgaacaugauccagaC.......................................................................................................................................................... 12     1    LCK-3
.........................................................................ugcugucgaacaugauccagaU.......................................................................................................................................................... 3      1    JCK-3
.........................................................................ugcugucgaacaugauccagaU.......................................................................................................................................................... 1      1    JCK-2
.........................................................................ugcugucgaacaugauccagaC.......................................................................................................................................................... 2      1    LT-3
.........................................................................ugcugucgaacaugauccagaC.......................................................................................................................................................... 3      1    LT-1
```

Mature (uggaucuuguuugacagcacu) — Star (ugcugucgaacaugauccaga)

cccaccucucuuguuuaccuucuggaucuuguuugacagcacucccaacguuguuguuuggaugacauagaagugcugucgaacaugauccagaaggugcugucgaacaacauacaaaaaggggguuuacuuuacguaccgcccaucacgaaaaaaaaaaaaaaauuguuuaauauuauuucaagauuuauuugaaugaaaauagaacagcuauuccuugguuucuuuaauuacuaaaacccuaugaucuua

ugcugucgaacaugauccagaU	1	1	LT-1
ugcugucgGacaugauccagaa	14	1	JCK-3
ugcugucgaacaugauccagaU	3	1	LT-2
ugcugucgaacaugauccagaa	3	0	LT-3
ugcugucgaacaugauccagaa	1	0	JCK-1
ugcugucgaacaugauccagaC	8	1	LT-2
ugcugucgaacaugauccagaC	1	1	JCK-3
ugcugucgaCcaugauccagaa	1	1	LT-1
ugcugucgaacaugauccagaa	6	0	LT-1
ugcugucgaacaugauccagaagg	1	0	JCK-3
ugcugucgaacaugauccagaagg	2	0	JCK-2
gcugucgaacaugauccag	6	0	LCK-3
gcugucgGacaugauccaga	9	1	JCK-2
gcugucgGacaugauccaga	5	1	JCK-3
gcugucgGacaugauccaga	2	1	JCK-1
cugucgaacaugauccag	1	0	LT-3
cugucgaacaugauccaga	10	0	LT-3
cugucgaacaugauccaga	21	0	LT-2
cugucgaacaugauccaga	8	0	LCK-3
cugucgaacCugauccaga	1	1	LT-2
cugucgaacaugauccagU	1	1	LT-2
cugucgaacaugauccaga	3	0	JCK-2
cugucgCacaugauccaga	1	1	LT-1
cugucgaacaugauccaga	7	0	LT-1
cugucgaacaugauccagaU	7	1	LCK-3
cugucgaacaugauccagaa	1	0	JCK-3
cugucgaacaugauccagaa	1	0	LT-2
cugucgaacaugauccagaa	2	0	JCK-2
cugucgaacaugauccagaa	4	0	LT-1
cugucgaacaugauccagUa	2	1	LT-2
cugucgaacaugauccagaa	3	0	LT-3
cugucgaacaugauccagaa	4	0	LCK-3
cugucgaacaugauccagaU	1	1	LT-1
cugucgaacaugauccagaag	13	0	JCK-2
cugucgaacaugauccagaaA	1	1	JCK-2
cugucgaacaugauccagaag	9	0	JCK-1
cugucgaacaugauccagaag	21	0	LCK-3
cugucgaacaugauccagaaU	3	1	LT-1
cugucgaacaugauccagaag	12	0	LT-1
cugucgaacaAgauccagaag	9	1	LCK-3
cugucgCacaugauccagaag	1	1	LT-1
cugucgaacaugauccagaag	20	0	LT-3
cugucgaacaugauccagaag	10	0	LT-2
cugucgaacaugauccagaag	18	0	JCK-3
cugucgCacaugauccagaag	1	1	LT-2
cugucgaacaugauccagaagA	3	1	JCK-2
cugucgaacaugauccagaagUu	1	1	LT-3
cugucgaacaugauccagaagUu	2	1	LT-2
ugucgaacaugauccaga	2	0	LT-1
ugucgaacaugauccaga	1	0	LT-3
ugucgaacaugauccagaagg	5	0	LT-1
ugucgaacaugauccagaagg	2	0	JCK-3
ugucgaacaugauccagaagg	6	0	LT-2
ugucgaacaugauccagaagg	4	0	LT-3
ugucgaacaugauccagaagg	2	0	JCK-2
ucgaacaugauccagaag	1	0	LT-1
ucgaacaugauccagaagg	1	0	JCK-1
ucgaacaugauccagaaggu	1	0	LT-2
ucgaacaugauccagaaggug	27	0	LCK-1
ucgaacaugauccagaaggug	15	0	LT-3
ucgaacaugauccagaaggug	2	0	JCK-2
ucgaacaugauccagaaggug	1	0	JCK-1
ucgaacaugauccagaaggug	9	0	LT-2
ucgaacaugauccagaaggug	23	0	LCK-2
ucgaacaugauccagaagguA	1	1	LT-1
ucgaacaugauccagaaggug	16	0	LT-1
cgaacCugauccagaaggu	1	1	LT-3
cgaacaugauccagaaggu	2	0	LT-3
cgaacaugauccagaaggu	1	0	LT-2
cgaacaugauccagaaggug	1	0	JCK-2

Mature **Star**

cccaccucucuuguuuaccuucuggaucuuguuugacagcacucccaacguuguuguuuggaugacauagaagugcugucgaacaugauccagaaggugcugucgaacaacauacaaaaggguuuacuuuacguaccgcccaucacgaaaaaaaaaaaaaaauuguuuaauauuauuucaagauuuauuugaaugaaaauagaacagcuauuccuugguuucuuuaauuacuaaaacccuaugaucuua

cgaacaugauccagaaggug	3	0	LT-1
cgaacaugauccagaaggug	2	0	LT-3
cgaacaugauccagaaggugc	3	0	JCK-1
cgaacaugauccagaaggugc	6	0	LT-1
cgaacaugauccagaaggugc	4	0	JCK-3
cgaacaugauccagaaggugc	6	0	JCK-2
cgaacaugauccagaaggugc	3	0	LT-3
aacaugauccagaaggugcugucgaaca	2	0	JCK-3
acaugauccagaaggugcugucg	1	0	LT-3
acaAgauccagaaggugcugucga	1	1	JCK-2
acaAgauccagaaggugcugucga	2	1	JCK-1
acaAgauccagaaggugcugucga	2	1	JCK-3
acaugauccagaaggugcugucgaa	1	0	JCK-1
acaugauccagaaggugcugucgaac	2	0	JCK-1
acaugauccagaaggugcugucgaac	1	0	JCK-2
acaugauccagaaggugcugucgaac	1	0	JCK-3
caugauccagaaggugcugucgaaca	1	0	LT-1
uccagaaggugcugucgaaca	1	0	LT-3
uccagaaggugcugucgaaca	2	0	LT-1
uccagaaggugcugucgaaca	2	0	LT-2
uccagaaggugcugucgaacaa	3	0	LT-1
uccagaaggugcugucgaacaa	1	0	LT-3
uccagaaggugcugucgaacaac	4	0	LT-2
uccagaaggugcugucgaacaac	12	0	JCK-2
uccagaaggugcugucgaacaac	4	0	JCK-1
uccagaaggugcugucgaacaac	2	0	LT-1
uccagaaggugcugucgaacaac	8	0	LT-3
uccagaaggugcugucgaacaac	5	0	LCK-3
uccagaaggugcugucgaacaac	15	0	JCK-3
uccagaaggugcugucgaacaacU	6	1	LCK-3
uccagaaggugcugucgaacaacU	1	1	JCK-2
uccagaaggugcugucgaacaacU	1	1	LT-3
uccagaaggugcugucgaacaaca	3	0	LT-3
uccagaaggugcugucgaacaaca	2	0	JCK-3
uccagaaggugcugucgaacaacN	1	1	LT-2
uccagaaggugcugucgaacaacU	2	1	LT-1
uccagaaggugcugucgaacaaca	2	0	LT-2
agaaggugcugucgaaca	1	0	LT-3
agaUggugcugucgaacaaca	1	1	JCK-3
agaaggugcugucgaacaaca	4	0	LCK-3
agaUggugcugucgaacaaca	1	1	JCK-2
agaaggugcugucgaacaacau	1	0	LT-3
agaaggugcugucgaacaacau	1	0	LT-1
agaaggugcugucgaacaacaua	2	0	LT-1
agaaggugcugucgaacaacaua	2	0	LT-2
gaaggugcugucgaacaacauacaaaaag	1	0	JCK-1
gugcugucgaacaaGaua	2	1	LCK-3
gugcugucgaacaaGaua	1	1	JCK-2
